# Identification of late-stage tau accumulation using plasma phospho-tau217

**DOI:** 10.1016/j.ebiom.2024.105413

**Published:** 2024-11-04

**Authors:** Marcel S. Woo, Joseph Therriault, Erin M. Jonaitis, Rachael Wilson, Rebecca E. Langhough, Nesrine Rahmouni, Andrea Lessa Benedet, Nicholas J. Ashton, Cécile Tissot, Juan Lantero-Rodriguez, Arthur C. Macedo, Stijn Servaes, Yi-Ting Wang, Jaime Fernandez Arias, Seyyed Ali Hosseini, Tobey J. Betthauser, Firoza Z. Lussier, Robert Hopewell, Gallen Triana-Baltzer, Hartmuth C. Kolb, Andreas Jeromin, Eliane Kobayashi, Gassan Massarweh, Manuel A. Friese, Jesse Klostranec, Paolo Vilali, Tharick A. Pascoal, Serge Gauthier, Henrik Zetterberg, Kaj Blennow, Sterling C. Johnson, Pedro Rosa-Neto

**Affiliations:** a Institute of Neuroimmunology and Multiple Sclerosis, University Medical Centre Hamburg Eppendorf, 20251, Hamburg, Germany; b Department of Neurology, University Medical Centre Hamburg Eppendorf, 20251, Hamburg, Germany; c Translational Neuroimaging Laboratory, McGill Research Centre for Studies in Aging, Montreal, Quebec, QC H4H 1R3, Canada; d Department of Neurology and Neurosurgery, Faculty of Medicine, McGill University, Montreal, Quebec, QC H3A 1A1, Canada; e Wisconsin Alzheimer's Institute, University of Wisconsin School of Medicine and Public Health, Madison, WI, 53726, USA; f Wisconsin Alzheimer's Disease Research Centre, University of Wisconsin School of Medicine and Public Health, University of Wisconsin-Madison, Madison, WI, 53726, USA; g Department of Psychiatry and Neurochemistry, The Sahlgrenska Academy at the University of Gothenburg, S-431 80, Mölndal, Sweden; h Wallenberg Centre for Molecular and Translational Medicine, University of Gothenburg, 40530, Gothenburg, Sweden; i Molecular Biophysics and Integrated Bioimaging Department, Lawrence Berkeley National Laboratory, Berkeley, 510, CA, USA; j Department of Neurology and Psychiatry, University of Pittsburgh School of Medicine, Pittsburgh, PA, 15213, USA; k Neuroscience Biomarkers, Johnson and Johnson Medical Innovation (formerly Janssen Research & Development), La Jolla, CA, 92121, USA; l ALZpath. Inc, Carlsbad, CA, 92008, USA; m Montreal Neurologic Institute and Hospital, Department of Diagnostic and Interventional Neuroradiology, McGill University Health Centre, 3801 Rue University, Montréal, QC, H3A 2B4, Canada; n Clinical Neurochemistry Laboratory, Sahlgrenska University Hospital, S-431 80, Mölndal, Sweden; o Department of Neurodegenerative Disease, UCL Institute of Neurology, Queen Square, London, WC1E 6BT, UK; p UK Dementia Research Institute at UCL, London, WC1E 6BT, UK; q Hong Kong Centre for Neurodegenerative Diseases, Clear Water Bay, 518172 Hong Kong, China

**Keywords:** Alzheimer's disease, p-tau217, Prognosis, Blood biomarker

## Abstract

**Background:**

Blood-based disease staging across the Alzheimer's disease (AD) continuum holds the promise to identify individuals that profit from disease-modifying therapies. We set out to identify Braak V^+^ (Braak V and/or VI) tau PET-positive individuals within amyloid-β (Aβ)-positive individuals using plasma biomarkers.

**Methods:**

In this cross-sectional study, we assessed 289 individuals from the TRIAD cohort and 306 individuals from the WRAP study across the AD continuum. The participants were evaluated by amyloid-PET with [^18^F]AZD4694 or [^11^C]PiB and tau-PET with [^18^F]MK6240 and measured plasma levels included total tau, phospho-tau isoforms (pTau) pTau-181, pTau-217, pTau-231, and N-terminal tau (NTA-tau). We evaluated the performances of plasma biomarkers using different analytic platforms to predict Braak V^+^ positivity in Aβ+ individuals.

**Findings:**

Highest associations with Braak V^+^ tau positivity in Aβ+ individuals were found for plasma pTau-217+^Janssen^ (AUC [CI_95%_] = 0.97 [0.94, 1.0]) and ALZpath pTau-217 (AUC [CI_95%_] = 0.93 [0.86, 1.0]) in TRIAD. Plasma ALZpath pTau-217 separated Braak V^+^ tau PET-positive individuals in the WRAP longitudinal study (AUC [CI_95%_] = 0.97 [0.94, 1.0]).

**Interpretation:**

Thus, we demonstrate that using adjusted cut-offs, plasma pTau-217 identifies individuals with later Braak stage tau accumulation which will be helpful to stratify patients for treatments and clinical studies.

**Funding:**

This research is supported by the 10.13039/100012479Weston Brain Institute, 10.13039/501100000024Canadian Institutes of Health Research (CIHR) [MOP-11-51-31; RFN 152985, 159815, 162303], Canadian Consortium of Neurodegeneration and Aging (CCNA; MOP-11-51-31 -team 1), the Alzheimer's Association [NIRG-12-92090, NIRP-12-259245], 10.13039/100009408Brain Canada Foundation (CFI Project 34874; 33397), the Fonds de Recherche du Québec—Santé (FRQS; Chercheur Boursier, 2020-VICO-279314). P.R-N and SG are members of the CIHR-CCNA Canadian Consortium of Neurodegeneration in Aging. Colin J. Adair Charitable Foundation.

**Research in context**

**Evidence before this study**

Several studies have reported a high performance of plasma phosphorylated tau (pTau) to detect Alzheimer's disease (AD) pathophysiology. Recent trials with amyloid-β (Aβ) targeting antibodies have highlighted the need for a disease staging of biological AD severity to identify individuals who benefit from anti-Aβ treatments.

**Added value of this study**

We showed in two separate cohorts that blood pTau-217 had excellent performance to separate individuals with PET-confirmed tau accumulation in Braak stages V and/or VI from individuals with tau accumulation in lower Braak stages. This was achieved by using higher cut-offs as compared to predicting tau accumulation in Braak stages I-IV and was independent of pre-selecting individuals with PET-confirmed Aβ accumulation.

**Implications of all the available evidence**

The findings suggest that in addition to identifying AD pathophysiology, adjusted cut-offs for blood pTau-217 are suitable for disease staging of individuals with Aβ accumulation. Thus, blood pTau-217 can be used in the diagnostic workup of individuals with suspected neurodegenerative diseases and to stratify the eligibility of individuals for anti-Aβ treatments. It is important that the findings are further validated in real-world clinical setting with diverse populations and backgrounds.

## Introduction

Alzheimer's disease (AD) is characterised by the buildup of amyloid-β (Aβ) plaques and the accumulation of tau into neurofibrillary tangles (NFT), occurring years before dementia symptoms appear. *In vivo* biomarkers using amyloid-positron emission tomography (PET) and tau-PET,^1^ as well as cerebrospinal fluid (CSF) assessments of Aβ and phosphorylated tau (pTau)^2^ can be used to identify core neuropathologic features of AD in living humans. Recently, plasma measurements of pTau have demonstrated close associations with PET, CSF and neuropathologic assessments of AD pathology.

Several recent studies have provided evidence that plasma pTau-217 can reliably detect elevated Aβ pathology in both asymptomatic and symptomatic individuals.^3^, ^4^, ^5^, ^6^, ^7^, ^8^, ^9^ Moreover, some of these studies have also reported that plasma pTau-217 performs equivalently to CSF biomarkers for the detection of AD pathology using PET.^3^, ^4^, ^5^ While recent evidence suggests that plasma pTau biomarkers are closely associated with Aβ pathology,^6^^,^^10^^,^^11^ in Aβ-positive (A+) individuals with cognitive symptoms, pTau biomarkers are also associated with tau tangle pathology^12^^,^^13^ as Aβ deposition assessed by PET have often plateaued at this stage.^14^^,^^15^

In neuropathologic assessments of AD, the severity of tau tangle pathology is assessed using the Braak staging system, with later stages indicating more advanced disease.^16^^,^^17^ Identifying late-stage tau accumulation may have clinical utility by increasing confidence that a set of clinical symptoms is due to AD, and furthermore may aid in treatment decisions, as individuals with more advanced tau may have lower clinical benefit following Aβ plaque removal.^18^ Thus, a cost-effective and widely accessible disease staging of tau accumulation is warranted to identify eligible individuals for Aβ-targeting therapies. However, the gold standard for *in vivo* Braak staging is PET^19^ which is not widely available. In contrast, AD staging by plasma biomarkers^20^ holds the promise to stratify A+ individuals across different clinical settings. Thus, this study assessed the ability of plasma biomarkers in independent cohorts to identify late-stage NFT accumulation assessed by tau-PET.

## Methods

### Study participants: TRIAD cohort

The individuals that were enrolled in the Translational Biomarkers of Aging and Dementia (TRIAD) cohort^21^ underwent Aβ PET with [^18^F]AZD4694, tau PET with [^18^F]MK6240 and magnetic resonance imaging (MRI). The TRIAD cohort consists of a high proportion of individuals who were recruited from a specialized tertiary care memory clinic. The cognitively unimpaired individuals are adult volunteers from the community. Thus, the TRIAD cohort consists of a greater proportion of individuals with cognitive impairment than WRAP. The TRIAD cohort included 277 Asian or white non-Hispanic individuals, and 12 members of an underrepresented group (Hispanic, African American, native American). Participants had a detailed clinical and cognitive assessment, including the Clinical Dementia Rating (CDR) and Mini-Mental State Examination (MMSE). In the TRIAD cohort, cognitively unimpaired individuals had no objective cognitive impairment, a CDR score of 0, and were asked to report any subjective cognitive decline in a questionnaire given during screening. Individuals with MCI had cognitive impairment, relatively preserved activities of daily living, and a CDR score of 0.5. Patients with Mild-to-moderate Alzheimer's clinical syndrome dementia had a CDR score between 0.5 and 2 and met the National Institute on Aging—Alzheimer's Association (NIA-AA) criteria for probable AD determined by a dementia specialist.^21^^,^^22^ Exclusion criteria for TRIAD were active substance abuse, recent head trauma, recent major surgery, or MRI/PET safety contraindications.^23^ All participants where the respective blood biomarkers, Aβ PET and tau PET were available were included for this study. Sex was self-reported by study participants.

### Study participants: WRAP cohort

The Wisconsin Registry for Alzheimer's Prevention (WRAP) participants included in this analysis underwent Aβ PET with [^11^C]PiB and tau-PET with [^18^F]MK6240. The WRAP study is a longitudinal observational study of individuals who at baseline do not have cognitive impairment and are between the ages of 40 and 65 at baseline. The cognitive status in WRAP was determined via a consensus review process using similar criteria as for TRIAD and included the CDR and MMSE. Furthermore, the cognitively unimpaired group was further partitioned into stable and subclinical decline subsets (for a comprehensive description of WRAP cognitive status determination and study inclusion and exclusion criteria, please see^24^). The WRAP cohort included 290 Asian or white non-Hispanic individuals, and 16 members of an underrepresented group (Hispanic, African American, native American). All participants where the respective blood biomarkers, Aβ PET and tau PET were available were included for this study. Sex was self-reported by study participants.

### MRI acquisition and processing

For TRIAD participants structural MRI data were acquired at the Montreal Neurological Institute (MNI) for all participants on a 3T Siemens Magnetom scanner using a standard head coil. Hippocampal volume was assessed using FreeSurfer version 6.0 and the Desikian–Killiany–Tourville atlas grey matter segmentation. WRAP T1-weighted structural MRI were acquired on a 3T GE Signa 750 and ROI- and tissue class segmented using SPM12. ROIs for PET analysis were generated by inverse warping AAL (for PiB) and Harvard–Oxford (for MK-6240) atlases to subject space using the deformation fields from the SPM12 unified segmentation and restricting to P_GM_ >0.3 as previously described.^25^ Hippocampal volumes were segmented using FSL FIRST^26^ applied to T1-weighted MRI. All imaging outcomes underwent routine visual quality assessment under supervision (TJB, SCJ).

### PET acquisition and processing

TRIAD participants had a T1-weighted MRI, and [^18^F]AZD4694 PET and [^18^F]MK6240 PET scans were acquired using a brain-dedicated Siemens high-resolution research tomograph. [^18^F]MK6240 PET images were acquired at 90–110 min after the intravenous bolus injection of the radiotracer and reconstructed using an ordered subset expectation maximization algorithm on a 4D volume with four frames (4 × 300 s), as previously described.^27^ [^18^F]AZD4694 PET images were acquired at 40–70 min after the intravenous bolus injection of the radiotracer and reconstructed with the same ordered subset expectation maximization algorithm on a 4D volume with three frames (3 × 600 s).^21^ A 6 min transmission scan with a rotating ^137^Cs point source was conducted at the end of each PET acquisition for attenuation correction. Images were corrected for motion, decay, dead time and random and scattered coincidences. In summary, PET images were linearly registered to T1-weighted image space, and the T1-weighted images were linearly and nonlinearly registered to the Alzheimer's Disease Neuroimaging Initiative (ADNI) reference space. To minimize the influence of meningeal spillover into adjacent brain regions, [^18^F]MK6240 images were skull-stripped in T1 space before transformations and blurring.^23^ The PET images in T1-space were linearly and nonlinearly registered to the ADNI space using transformations from the T1-weighted image to ADNI space. [^18^F]MK6240 standardized uptake value ratio (SUVRs) were calculated using the cerebellar crus I grey matter as a reference region,^23^^,^^28^ as derived from the SUIT cerebellum atlas.^29^ [^18^F]AZD4694 SUVRs were calculated using the whole cerebellum grey matter as the reference region. PET images were spatially smoothed to achieve an 8-mm full-width at half-maximum resolution. The global [^18^F]AZD4694 SUVR composite included the precuneus, prefrontal, orbitofrontal, parietal, temporal and cingulate cortices.^29^ PET Braak-like stage segmentation was previously described.^23^^,^^30^ Stages included the following regions: Braak I (transentorhinal), Braak II (entorhinal and hippocampus), Braak III (amygdala, para-hippocampal gyrus, fusiform gyrus and lingual gyrus), Braak IV (insula, inferior temporal, lateral temporal, posterior cingulate and inferior parietal), Braak V (orbitofrontal, superior temporal, inferior frontal, cuneus, anterior cingulate, supramarginal gyrus, lateral occipital, precuneus, superior parietal, superior frontal and rostromedial frontal) and Braak VI (paracentral, postcentral, precentral and pericalcarine).^16^^,^^31^ Tau PET positivity for each Braak stage was defined as [^18^F]MK6240 SUVR >2.5 standard deviations of the mean of the cognitively unimpaired and amyloid-negative participants. We used [^18^F]AZD4694^21^ to determine Centiloids and used a cut-off >20 to determine off Aβ positivity. The classification into Braak I+ or Braak V+ participants was based on PET-based tau positivity in the highest Braak stage. Thus, Braak I+ participants were defined as participants with tau accumulation above the threshold in Braak stages I or higher. Braak V+ positive participants were defined as participants with tau accumulation above the threshold in Braak stages V or VI regardless of PET-based tau positivity in earlier Braak stages.

WRAP participants underwent [^11^C]PiB amyloid and [^18^F]MK6240 tau PET imaging on either an ECAT EXACT HR+ or Siemens Biograph Horizon tomograph as previously described.^25^^,^^32^^,^^33^ Reconstructed dynamic PiB PET images were smoothed (3 mm Gaussian), inter-frame aligned, dynamically denoised (HighlY constrained backPRojection, HYPR), and registered to T1-w MRI. Cortical PiB distribution volume ratio (DVR) was calculated using graphical analysis (cerebellum GM reference region, k_2_’ = 0.149 min^−1^, t∗ = 35 min)^34^^,^^35^ from eight bi-lateral ROIs.^36^ A 20-Centiloid equivalent cutoff of global PiB DVR ≥1.18 was used following Betthauser and colleagues.^37^^,^^38^ Late-frame dynamic [^18^F]MK6240 reconstructed images (5 min frame ×4) were smoothed (6 mm Gaussian), interframe aligned, summed 70–90 min post-injection and registered to T1-weighted MRI (SPM12). SUVR was calculated using inferior cerebellar GM as a reference region.^25^ Regional SUVR for regions corresponding to Braak neurofibrillary tangle staging were extracted from the Harvard–Oxford atlas. Tau PET positivity for each Braak stage was defined as [^18^F]MK6240 SUVR >2.5 standard deviations above the mean value among cognitively unimpaired, amyloid-negative participants.

### Fluid biomarkers

Plasma samples were collected at the screening visit which generally precedes the first PET visit by approximately 1–2 months according to standard procedures in the clinical routine.^14^ Samples were then rapidly frozen for long-term storage at −80 °C.^14^ The Quanterix Single molecule array (Simoa) HD-X platform was used to quantify different tau species in plasma. pTau-181 and pTau-231 were quantified using in-house developed assays as previously described.^14^ Two commercially available pTau217 assays were evaluated: the Janssen R&D pTau-217^+^ assay^39^ and the ALZpath pTau217 assay.^9^ Plasma NTA-tau concentrations were quantified using an in house-developed Simoa immunoassay at the Clinical Neurochemistry Laboratory (Mölndal, Sweden).^40^ The measurements of the different biomarkers for both sites have been described in detail elsewhere.^9^ One technical replicate was measured per sample. For the TRIAD cohort the following analytes were measured: pTau-181, pTau-231, pTau-217^+^ Janssen, ALZpath pTau-217. All plasma analytes were measured at the Department of Psychiatry and Neurochemistry, University of Gothenburg except for pTau-217^+^ Janssen that was measured at Janssen R&D. For the WRAP cohort the following analytes were measured: pTau-181, pTau-231, ALZpath pTau-217. All plasma analytes were measured at the Department of Psychiatry and Neurochemistry, University of Gothenburg except for pTau-181 Janssen which was quantified by the commercial pTau181 Advantage V2.1 Simoa (#104111, Quanterix).

### Ethics

TRIAD was approved by the MNI PET working committee and the Douglas Mental Health University Institute Research Ethics Board (MP-18-2017-157). Written informed consent was obtained for all participants. WRAP was approved by the University of Wisconsin–Madison Health Sciences IRB (IRB00000366), and all participants provided written informed consent.

### Statistics

All analyses were performed using *R* within the *R Studio* environment. Sample size determination, and blinding were not performed. No specific exclusion criteria were defined for this study. For comparing plasma biomarker levels in TRIAD and WRAP, we first tested for normality distribution with the Shapiro-Wilk test. Since our data was not normally distributed, we used the non-parametric Wilcoxon rank sum test between all conditions with Holm-Bonferroni correction for multiple comparisons. The mean differences and 95% confidence intervals were additionally reported. ROC analyses and estimation of area under the curve (AUC) and 95% confidence intervals (CInt) were calculated using the DeLong's method with the *pROC* package.^41^ We included biological sex, age, and *APOE* ε4 carriership as covariates for the ROC analyses. ROC curves were statistically compared using the DeLong's test with FDR-correction for multiple comparisons. In separate analyses for each cohort, we examined ROC characteristics of each plasma biomarker for separating the following conditions: T^Braak I+^ vs. T-;T^Braak V+^ vs. T- and T^Braak I–IV^ combined in all participants; T^Braak V+^ vs. T- and T^Braak I–IV^ in the Aβ+ subset of participants. For the TRIAD cohort, we additionally performed the following analyses: demented vs not-demented; A+T- vs. A-T-; A+T+ vs. A-T-; A+T+ vs. A+T- in cognitively unimpaired participants; and T^Braak V+^ vs. T- and T^Braak I–IV^ in the cognitively impaired Aβ+ subset of participants. The exact numbers for the subset comparisons are reported in the respective results sections and figure legends. Sensitivity, specificity, and accuracy as well as their 95% confidence intervals of continuous biomarker values to evaluate their performances were calculated using Youden's index. For the TRIAD cohort we additionally calculated Pearson correlation analyses of the blood biomarkers with the hippocampal volume separately for A-T-, A+T-, A+T^Braak I–IV^, and A+T^Braak V+^. The regression lines were fitted using the ordinary least square method. To compare the performance of pTau-217 alone or in combination with pTau-181 or pTau-231 we performed Cohen's Kappa agreement analysis.^42^ Therefore, we dichotomized the either all or A+ participants into T^Braak I+^ or T^Braak V+^ or the respective rest using the PET-based classification as gold standard and the biomarker-based classification using our determined cut-offs for each respective tau analyte and comparison. For testing the prediction of biomarkers combinations, participants needed to be classified as positive for one of the biomarkers. We reported Cohen's Kappa and the 95% confidence intervals. The Cohen's Kappa can be interpreted by: Cohen's Kappa ≥0.8 = almost perfect, ≥0.6 = substantial, ≥0.4 = moderate, ≥0.2 = fair, ≥0 = slight, <0 = poor agreement.^43^ For biomarker concentrations in Table 3, we reported mean with standard deviation (SD) when mean/SD >2 or median with interquartile range when mean/SD <2.

### Role of funders

This research is supported by the Weston Brain Institute, Canadian Institutes of Health Research, Canadian Consortium of Neurodegeneration and Aging, the Alzheimer's Association, Brain Canada Foundation, the Fonds de Recherche du Québec—Santé and the Colin J. Adair Charitable Foundation. None of the funders had a role in the study design, data collection, data analyses, data interpretation or writing.

## Results

### Study population TRIAD cohort

We included 289 participants from the TRIAD cohort (*n* = 174 cognitively unimpaired, *n* = 74 with mild cognitive impairment (MCI), *n* = 41 with Alzheimer's disease (AD)) who were separated according to brain Aβ status and tau accumulation in different Braak stages by [^18^F]AZD4694 PET and [^18^F]MK6240 PET respectively. TRIAD sample characteristics are summarized in Table 1 (demographics separated by sex are shown in Supplementary Table S1). We identified 166 (57%) participants without brain amyloidosis or tau pathology (A-T-, 60% female, mean age 60 years), 26 (9%) participants with amyloid aggregation without detectable tau accumulation by PET (A+T-, 54% female, mean age 72 years), 39 (14%) participants with brain amyloidosis and tau accumulation in Braak stages I–IV (A+T^Braak I–IV^, 67% female, mean age 72 years), and 58 (20%) participants with Aβ accumulation in the brain and tau accumulation in Braak stages V or VI (A+T^Braak V+^, 57% female, mean age 69 years).

**Table 1:** Patient characteristics of TRIAD.

	A-T-	A+T-	A+T^Braak I–IV^	A+T^Braak V+^
N (%)	166 (57.4)	26 (9.0)	39 (13.5)	58 (20.1)
N female, (%)	100 (60.2)	14 (53.8)	26 (66.7)	33 (56.9)
Diagnosis, Cognitively unimpaired, N (%)	137 (82.5)	16 (61.5)	20 (51.3)	1 (1.7)
Diagnosis, Mild cognitive impairment, N (%)	29 (17.5)	10 (38.5)	15 (38.5)	20 (34.5)
Diagnosis, Dementia, N (%)	0 (0.0)	0 (0.0)	4 (10.3)	37 (63.8)
*APOE* ε4 carriership, N (%)	44 (26.5)	5 (19.2)	18 (46.2)	44 (75.9)
Age (years), mean (SD)	59.5 (19.9)	71.9 (7.0)	72.3 (9.2)	68.9 (8.8)
Mini-Mental State Exam, mean (SD)	28.8 (2.1)	27.6 (4.6)	28.2 (2.4)	22.8 (5.7)
Educational years, mean (SD)	15.2 (3.6)	14.3 (3.1)	14.9 (3.6)	14.8 (3.5)
Total tau SUVR, mean (SD)	0.9 (0.1)	0.9 (0.1)	1.0 (0.1)	2.0 (0.9)
Total amyloid SUVR, mean (SD)	1.3 (0.1)	1.8 (0.4)	2.1 (0.4)	2.4 (0.5)
Hippocampal volume, mean, cm^3^, (SD)	3.6 (0.5)	3.4 (0.4)	3.4 (0.4)	3.0 (0.5)

### Study population WRAP cohort

Furthermore, we included 306 participants from the WRAP study (*n* = 296 cognitively unimpaired, *n* = 9 with MCI, *n* = 1 with dementia) who were assessed with [^18^F]MK6240 PET, including 306 who were also assessed with [^11^C]PiB PET. WRAP sample characteristics are shown in Table 2 (demographics separated by sex are shown in Supplementary Table S1). Among those with both amyloid and tau PET, we identified 209 (68%) A-T- participants (67% female, mean age 66 years), 44 (14%) A+T- participants (59% female, mean age 69 years), 27 (9%) A+T^Braak I–IV^ participants (67% female, mean age 69 years), 9 (3%) A+T^Braak I–IV^ participants (89% female, mean age 69 years), 15 (5%) A-T^Braak V+^ participants (80% female, mean age 68 years), and 2 (<1%) A-T^Braak V+^ participants (100% female, mean age 57 years).

**Table 2:** Patient characteristics of WRAP longitudinal study cohort.

	A-T-	A+T-	A+T^Braak I–IV^	A+T^Braak V+^	A-T^Braak I–IV^	A-T^Braak V+^
N (%)	209 (68.3)	44 (14.4)	27 (8.8)	9 (2.9)	15 (4.9)	2 (0.7)
N female (%)	139 (66.5)	26 (59.1)	18 (66.7)	8 (88.9)	12 (80.0)	2 (100.0)
Diagnosis, Cognitively unimpaired, N (%)	208 (99.5)	42 (95.5)	24 (88.9)	5 (55.6)	15 (100.0)	2 (100.0)
Diagnosis, Mild cognitive impairment, N (%)	1 (0.5)	2 (4.5)	3 (11.1)	3 (33.3)	0 (0.0)	0 (0.0)
Diagnosis, Dementia, N (%)	0 (0.0)	0 (0.0)	0 (0.0)	1 (11.1)	0 (0.0)	0 (0.0)
*APOE* ε4 carriership, N (%)	62 (29.7)	26 (59.1)	20 (74.1)	6 (66.7)	6 (40.0)	1 (50.0)
Age (years), mean (SD)	65.7 (6.7)	69.0 (5.4)	69.1 (6.3)	69.0 (4.1)	68.3 (7.0)	57.4 (2.9)
Mini-Mental State Exam, mean (SD)	29.5 (0.7)	29.1 (1.1)	28.9 (1.3)	27.0 (4.2)	29.5 (0.6)	29.0 (0.0)
Educational years, mean (SD)	16.2 (2.7)	16.5 (2.4)	16.4 (2.5)	15.1 (2.6)	15.8 (2.7)	18.0 (0.0)
Total tau SUVR, mean (SD)	1.0 (0.1)	1.0 (0.1)	1.1 (0.1)	1.8 (0.4)	1.1 (0.1)	1.4 (0.0)
Total amyloid DVR, mean (SD)	1.1 (0.1)	1.4 (0.2)	1.5 (0.2)	1.8 (0.2)	1.1 (0.1)	1.1 (0.0)
Hippocampal volume, mean, cm^3^, (SD)	3.8 (0.4)	3.8 (0.4)	3.6 (0.4)	3.5 (0.6)	3.6 (0.4)	3.8 (0.3)

### Blood pTau levels across the Braak stages

**Fig. 1:**
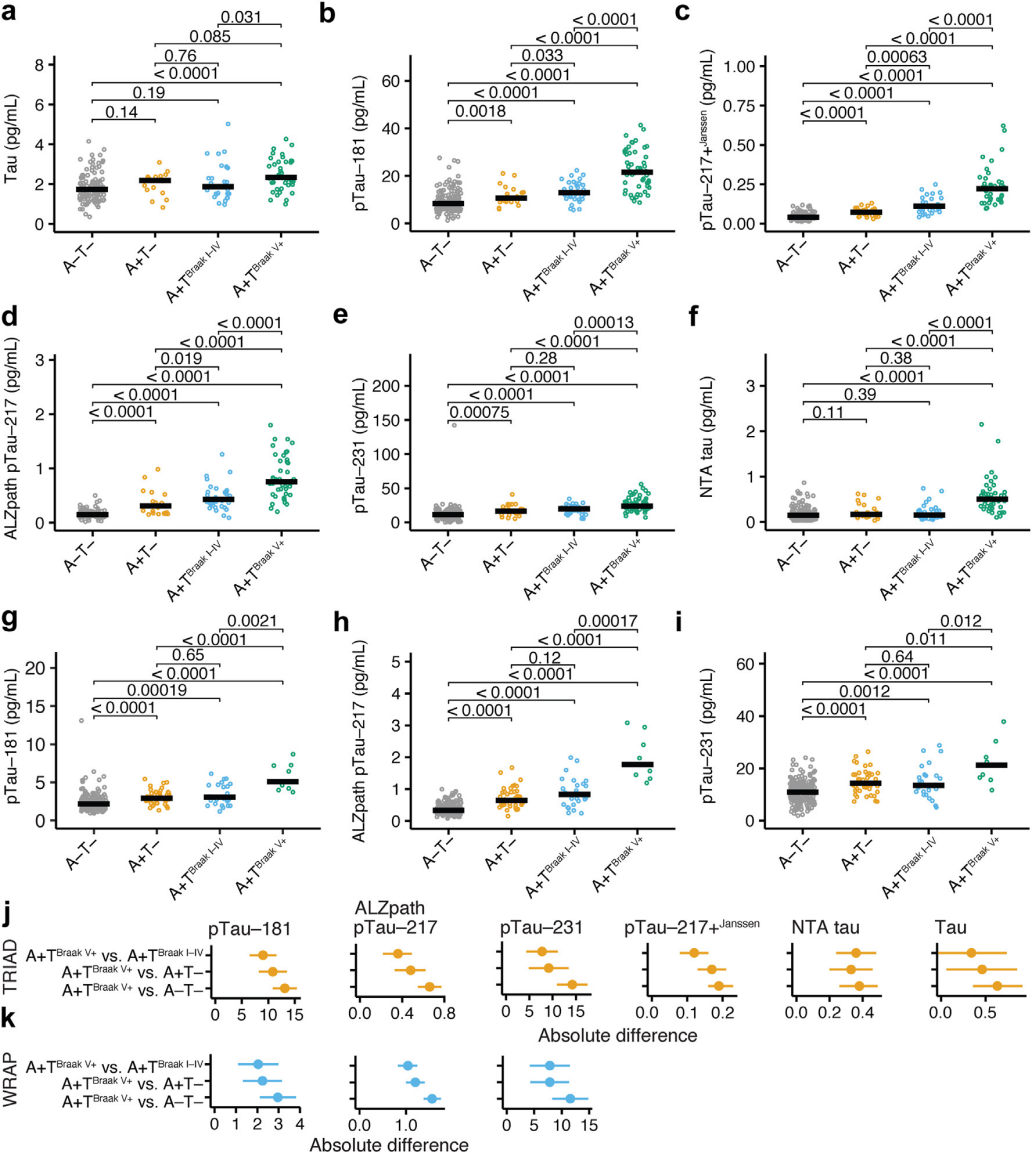
**Blood tau biomarkers across different Braak stages**. (a–f) **TRIAD cohort** plasma levels of (a) total tau (pg/mL), (b) pTau-181 (ng/mL), (c) pTau-217+^Janssen^ (pg/mL), (d) ALZpath pTau-217 (pg/mL), (e) pTau-231 (pg/mL), and (f) NTA tau (pg/mL) in individuals without amyloid and tau pathology (A-T-, *n* = 166), in individuals with amyloid accumulation and no tau accumulation (A+T-, *n* = 26), amyloid accumulation and tau PET positivity in Braak stages I-IV (A+T^Braak I–IV^, *n* = 39), and amyloid accumulation and tau PET positivity in Braak stages V and VI (A+T^Braak V+^, *n* = 58). (g–i) **WRAP cohort** plasma levels of (g) pTau-181 (ng/mL), (h) ALZpath pTau-217 (pg/mL), and (i) pTau-231 (pg/mL) in individuals without amyloid and tau pathology (A-T-, *n* = 209), in individuals with amyloid accumulation (A+T-, *n* = 44), amyloid accumulation and tau PET positivity in Braak stages I-IV (A+T^Braak I–IV^, *n* = 27), and amyloid accumulation and tau PET positivity in Braak stages V and VI (A+T^Braak V+^, *n* = 9). Individual data points and the median are shown. (j) Mean differences and respective 95% confidence intervals for comparisons between A+T^Braak V+^ and A-T-, A+T^Braak V+^ and A+T-, and A+T^Braak V+^ and A+T^Braak I–IV^ in the **TRIAD cohort**. (k) Mean differences and respective 95% confidence intervals for comparisons between A+T^Braak V+^ and A-T-, A+T^Braak V+^ and A+T-, and A+T^Braak V+^ and A+T^Braak I–IV^ in the **WRAP cohort**. Bonferroni-Holmes adjusted Wilcoxon rank sum test was used for statistical comparisons. The exact *P*-values are provided in the figure. pTau, phospho-tau; NTA tau, N-terminal tau.

First, we analysed the blood levels of total tau (Fig. 1a), pTau-181 (Fig. 1b), pTau-217+^Janssen^ (Fig. 1c), ALZpath pTau-217 (Fig. 1d), pTau-231 (Fig. 1e), and NTA-tau (Fig. 1f) in A-T-, A+T-, A+T^Braak I-IV^, and A+T^Braak V+^ participants of the TRIAD cohort.

We observed a significant stepwise increase in pTau-217+^Janssen^, and ALZpath pTau-217 across the four A/T categories. pTau-181 and pTau-231 levels were increased in patients with A+T- in comparison to A-T-, and in A+T^Braak V+^ against all other groups. However, we did not find significant differences between A+T- and A+T^Braak I–IV^ for pTau-181 and pTau-231. Blood levels of total tau was significantly increased in A+T^Braak V+^ participants in comparison to A+T- participants, and blood levels of NTA-tau were increased in A+T^Braak V+^ participants in comparison to all other groups (descriptive statistics of all blood biomarkers are provided in Table 3). We replicated this analysis in WRAP for analytes that were available (pTau-181, ALZpath pTau-217, and pTau-231). We again observed stepwise increases for pTau-217 along the AD continuum (A-T-, A+T-, A+T^Braak I-IV^, A+T^Braak V+^). Results were similar but weaker for pTau-181 and pTau-231 (Fig. 1g–i).

**Table 3:** Blood biomarkers levels in the TRIAD and WRAP cohorts.

Analyte	Cohort	A-T-	A+T-	A+T^Braak I–IV^	A+T^Braak V+^
		Mean (SD)∗/Median (IQR)^#^	Mean (SD)∗/Median (IQR)^#^	Mean (SD)∗/Median (IQR)^#^	Mean (SD)∗/Median (IQR)^#^
Total tau (pg/mL)	TRIAD	1.84 (0.68)∗	2.01 (0.61)∗	2.13 (0.94)∗	2.47 (0.79)∗
pTau-181 (pg/mL)	TRIAD	9.28 (4.49)∗	11.55 (3.79)∗	13.42 (4.043)∗	22.41 (8.11)∗
pTau-217+^Janssen^ (pg/mL)	TRIAD	0.052 (0.021)∗	0.073 (0.031)∗	0.12 (0.052)∗	0.24 (0.12)∗
ALZpath pTau-217 (pg/mL)	TRIAD	0.16 (0.071)∗	0.31 (0.18–0.38)^#^	0.43 (0.32–0.56)^#^	0.83 (0.37)∗
pTau-231 (pg/mL)	TRIAD	11.34 (7.61–15.55)^#^	17.57 (8.091)∗	19 (6.33)∗	26.74 (10.23)∗
NTA tau (pg/mL)	TRIAD	0.15 (0.075–0.25)^#^	0.17 (0.12–0.32)^#^	0.15 (0.10–0.24)^#^	0.50 (0.35–0.63)^#^
pTau-181 (pg/mL)	WRAP	2.42 (1.17)∗	3.07 (0.92)∗	3.34 (1.34)∗	5.69 (1.76)∗
ALZpath pTau-217 (pg/mL)	WRAP	0.37 (0.16)∗	0.73 (0.31)∗	0.83 (0.58–1.14)^#^	1.97 (0.69)∗
pTau-231 (pg/mL)	WRAP	11.04 (4.10)∗	14.91 (4.60)∗	14.79 (6.10)∗	21.98 (8.11)∗

Abbreviations: pTau, phospho-tau; SD, standard deviation; IQR, interquartile range; ∗mean (SD) is provided; ^#^median (IQR) is provided, See Statistics section for justification of descriptive parameters.

In the next step, we calculated the absolute mean differences for A+T^Braak V+^ vs. A-T-, A+T^Braak V+^ vs. A+T- and A+T^Braak V+^ vs. A+T^Braak I-IV^. Our analyses confirmed stepwise increases of the mean differences for pTau-217+^Janssen^ and ALZpath pTau-217 in TRIAD when contrasting A+T^Braak V+^ with A+T^Braak I-IV^, A+T-, and A-T- individuals (Fig. 1j). This was mirrored in the WRAP cohort, where ALZpath pTau-217 showed a gradual increase of the mean differences for the same comparisons (Fig. 1k). We concluded that across both cohorts, the gradual PET-based classification was represented by pTau-217+^Janssen^ and ALZpath pTau-217. In addition, we analysed the relationship between the different tau analytes and neurodegeneration, another hallmark of AD. Therefore, we calculated the correlation between the hippocampal volume and the tau analytes separately in A-T-, A+T-, A+T^Braak I–IV^, and A+T^Braak V+^ TRIAD participants (Supplementary Figure S1, Supplementary Table S2) where we did not detect a significant association between the different pTau-217 assays and hippocampal degeneration. This underlines that pTau-217 reflects tau and amyloid brain accumulation in the AD continuum.

### pTau-217 identifies participants with tau accumulation in late Braak stages

**Fig. 2:**
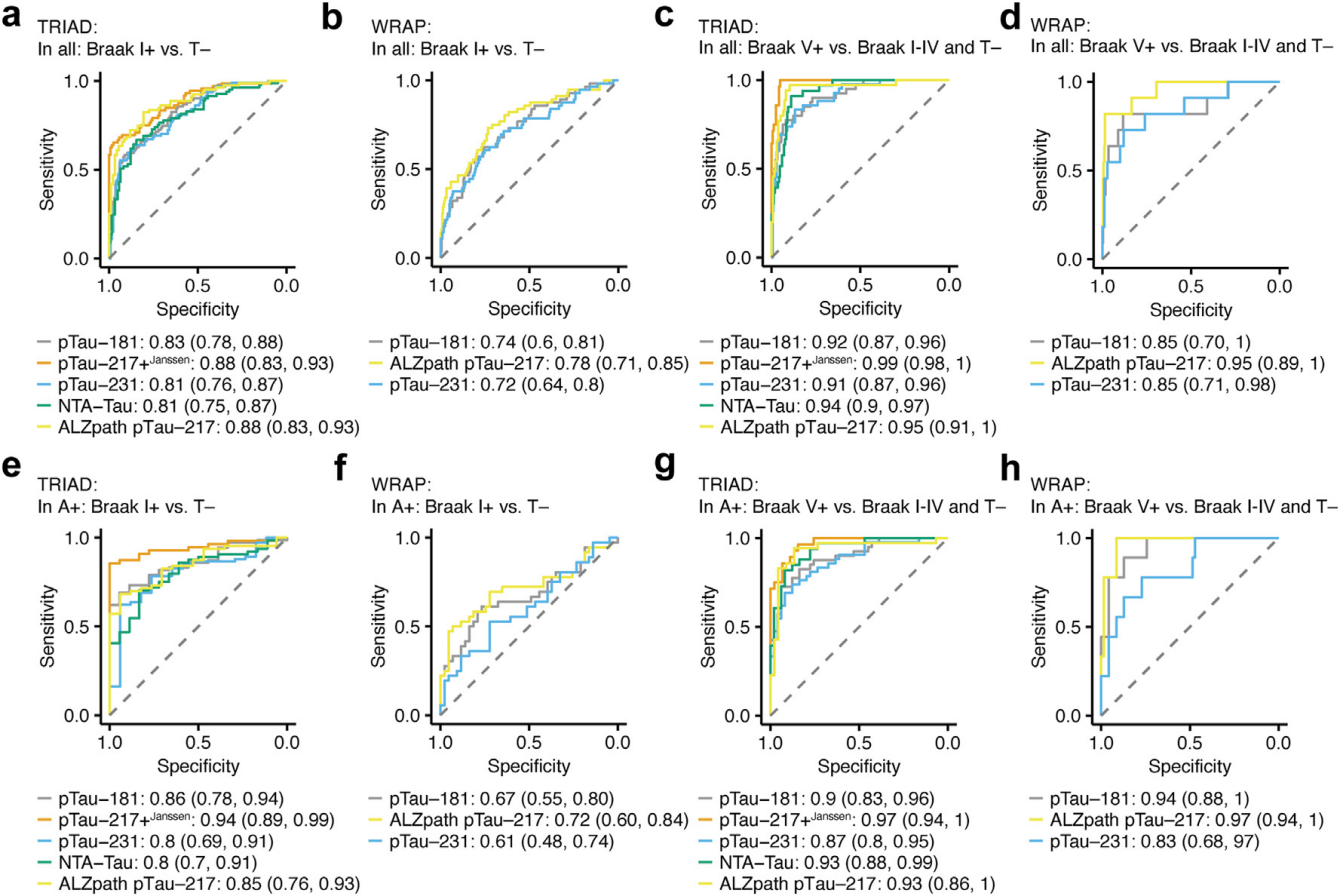
**Discrimination of tau accumulation in different Braak stages by blood tau biomarkers in TRIAD and WRAP**. Receiver operating characteristic (ROC) analyses to identify T^Braak I+^ individuals without preselection in TRIAD (a) and WRAP (b), T^Braak V+^ individuals without preselection in TRIAD (c) and WRAP (d), T^Braak I+^ individuals in Aβ+ participants in TRIAD (e) and WRAP (f), T^Braak V+^ individuals in Aβ+ participants in TRIAD (g) and WRAP (h). pTau-181 (pg/mL), pTau-217+^Janssen^ (pg/mL), ALZpath pTau-217 (pg/mL), pTau-231 (pg/mL), and NTA tau (pg/mL) were tested in TRIAD. pTau-181 (pg/mL), ALZpath pTau-217 (pg/mL), pTau-231 (pg/mL) were tested in WRAP. Area under the curve (AUC) and confidence intervals are shown in the figure. The ROC models include biological sex, age, and APOE ε4 status as covariates. Area under the curves (AUC) and 95% confidence intervals are shown. Dashed line represents AUC of 0.5. pTau, phospho-tau; NTA tau, N-terminal tau.

Next, we performed receiver operating characteristic (ROC) analysis of pTau-181, pTau-217+^Janssen^, ALZpath pTau-217, and NTA-tau to discriminate tau accumulation. In the first step, we aimed to identify AD vs. non-AD, A+T- vs. A-T-, and A+T+ vs. A-T- in the TRIAD cohort (Supplementary Figure S2). Only the pTau-217 assays showed AUCs >0.9 for all three comparisons (AD vs. all: pTau-217+^Janssen^, AUC = 0.95; ALZpath pTau-217, AUC = 0.92; A+T-vs. A-T-: pTau-217+^Janssen^, AUC = 0.9; ALZpath pTau-217, AUC = 0.92; A+T+ vs. A-T-: pTau-217+^Janssen^, AUC = 1.0; ALZpath pTau-217, AUC = 0.99; the results of the statistical comparisons are provided in Supplementary Table S3). In the next step, our goal was to determine tau accumulation in different Braak stages in the TRIAD and WRAP cohorts. Therefore, in separate analyses for each cohort, we examined ROC characteristics of each plasma biomarker for separating the following conditions: participants with any tau accumulation (T^Braak I+^) vs. participants without tau accumulation (T-); T^Braak V^+ vs. T- and T^Braak I–IV^ combined in all participants, and in the Aβ+ subset of participants. To separate T^Braak I+^ from T- in all individuals, we found the highest AUCs for pTau-217+^Janssen^ and ALZpath pTau-217 in TRIAD (Fig. 2a; pTau-217+^Janssen^, AUC = 0.88; ALZpath pTau-217, AUC = 0.88), and WRAP (Fig. 2b; ALZpath pTau-217, AUC = 0.78). Similarly, pTau-217 best distinguished T^Braak V+^ individuals in TRIAD (Fig. 2c; pTau-217+^Janssen^, AUC = 0.99; ALZpath pTau-217, AUC = 0.95) and WRAP (Fig. 2d; ALZpath pTau-217, AUC = 0.95). Additionally, pTau-181 (AUC = 0.92), pTau-231 (AUC = 0.91), and NTA-tau (AUC = 0.94) identified T^Braak V+^ individuals with an AUC >0.9 in TRIAD.

Next, we repeated the analyses in each cohort using the subset of Aβ+ participants. The best separation to identify T^Braak I+^ individuals among Aβ+ participants (i.e., relative to A+T-; Fig. 2e) was achieved using pTau-217+^Janssen^ (AUC = 0.94) and ALZpath pTau-217 (AUC = 0.85), and pTau-181 (AUC = 0.86) in TRIAD. In the parallel WRAP analyses, all analytes showed AUCs <0.8 (Fig. 2f). When examining A+T^Braak V+^ vs the remaining Aβ+ participants in TRIAD, the identification of A+T^Braak V+^ (Fig. 2g) was possible with an AUC >0.9 with pTau-181 (AUC = 0.9), pTau-217+^Janssen^ (AUC = 0.97), ALZpath pTau-217 (AUC = 0.93) and NTA-tau (AUC = 0.93). In parallel WRAP analyses, results with ALZpath pTau-217 were similar in Aβ+ participants (Fig. 2h; T^Braak I+^: AUC = 0.72; T^Braak V+^: AUC = 0.97), but in this group, pTau-181 also classified A+^Braak V+^ participants well (AUC = 0.94). Thus, blood levels of pTau and NTA-tau can be used to identify A+T^Braak V+^ individuals without preselection and in an Aβ+ enriched cohort (all AUCs and confidence intervals are provided in Table 4 for TRIAD and Table 5 for WRAP; the results of the statistical comparisons are provided in Supplementary Table S3; ROC analyses without inclusion of covariates are shown in Supplementary Figure S3).

**Table 4:** Accuracy analysis of blood biomarkers in TRIAD.

Analyte	Comparison	AUC (95% CInt)	Thr.	Sens. (95% CInt)	Spec. (95% CInt)	Acc. (95% CInt)
pTau-181 (pg/mL)	T^Braak V+^ vs. T- and T^Braak I–IV^	0.92 (0.87, 0.96)	17.26	0.83 (0.71, 0.93)	0.92 (0.78, 0.97)	0.9 (0.8, 0.94)
pTau-217+^Janssen^ (pg/mL)	T^Braak V+^ vs. T- and T^Braak I–IV^	0.99 (0.98, 1)	0.12	1 (1, 1)	0.93 (0.89, 0.97)	0.94 (0.91, 0.97)
ALZpath pTau-217 (pg/mL)	T^Braak V+^ vs. T- and T^Braak I–IV^	0.95 (0.91, 1)	0.45	0.95 (0.84, 1)	0.89 (0.82, 0.97)	0.9 (0.84, 0.96)
pTau-231 (pg/mL)	T^Braak V+^ vs. T- and T^Braak I–IV^	0.91 (0.87, 0.96)	22.06	0.82 (0.68, 0.98)	0.88 (0.64, 0.95)	0.87 (0.7, 0.91)
NTA tau (pg/mL)	T^Braak V+^ vs. T- and T^Braak I–IV^	0.94 (0.9, 0.97)	0.28	0.94 (0.8, 1)	0.82 (0.74, 0.92)	0.84 (0.77, 0.92)
pTau-181 (pg/mL)	T^Braak I+^ vs. T-	0.83 (0.78, 0.88)	11.38	0.72 (0.6, 0.86)	0.81 (0.64, 0.89)	0.77 (0.71, 0.82)
pTau-217+^Janssen^ (pg/mL)	T^Braak I+^ vs. T-	0.88 (0.83, 0.93)	0.09	0.73 (0.61, 0.84)	0.93 (0.85, 0.98)	0.85 (0.8, 0.9)
ALZpath pTau-217 (pg/mL)	T^Braak I+^ vs. T-	0.88 (0.83, 0.93)	0.32	0.77 (0.65, 0.86)	0.88 (0.77, 0.95)	0.83 (0.76, 0.88)
pTau-231 (pg/mL)	T^Braak I+^ vs. T-	0.81 (0.76, 0.87)	15.49	0.76 (0.55, 0.88)	0.75 (0.62, 0.91)	0.75 (0.69, 0.81)
NTA tau (pg/mL)	T^Braak I+^ vs. T-	0.81 (0.75, 0.87)	0.24	0.55 (0.34, 0.83)	0.8 (0.47, 0.94)	0.7 (0.61, 0.75)
pTau-181 (pg/mL)	A+T^Braak V+^ vs. A+T- or A+T^Braak I–IV^	0.9 (0.83, 0.96)	15.62	0.81 (0.62, 0.93)	0.86 (0.73, 0.98)	0.84 (0.76, 0.9)
pTau-217+^Janssen^ (pg/mL)	A+T^Braak V+^ vs. A+T- or A+T^Braak I–IV^	0.97 (0.94, 1)	0.13	0.97 (0.73, 1)	0.82 (0.67, 1)	0.88 (0.8, 0.93)
ALZpath pTau-217 (pg/mL)	A+T^Braak V+^ vs. A+T- or A+T^Braak I-IV^	0.87 (0.8, 0.95)	0.6	0.84 (0.7, 0.97)	0.84 (0.64, 0.96)	0.84 (0.76, 0.91)
pTau-231 (pg/mL)	A+T^Braak V+^ vs. A+T- or A+T^Braak I–IV^	0.93 (0.86, 1)	21.81	0.75 (0.52, 0.89)	0.82 (0.67, 0.96)	0.77 (0.7, 0.85)
NTA tau (pg/mL)	A+T^Braak V+^ vs. A+T- or A+T^Braak I–IV^	0.93 (0.88, 0.99)	0.27	0.94 (0.8, 1)	0.82 (0.69, 0.94)	0.87 (0.79, 0.93)
pTau-181 (pg/mL)	A+T^Braak I+^ vs. A+T-	0.86 (0.78, 0.94)	13.08	0.73 (0.55, 0.9)	0.89 (0.67, 1)	0.77 (0.63, 0.89)
pTau-217+^Janssen^ (pg/mL)	A+T^Braak I+^ vs. A+T-	0.94 (0.89, 0.99)	0.09	0.86 (0.61, 0.98)	0.94 (0.72, 1)	0.87 (0.71, 0.95)
ALZpath pTau-217 (pg/mL)	A+T^Braak I+^ vs. A+T-	0.8 (0.69, 0.91)	0.39	0.82 (0.54, 0.98)	0.76 (0.47, 1)	0.8 (0.61, 0.91)
pTau-231 (pg/mL)	A+T^Braak I+^ vs. A+T-	0.85 (0.76, 0.93)	20	0.7 (0.38, 0.89)	0.76 (0.47, 1)	0.71 (0.49, 0.84)
NTA tau (pg/mL)	A+T^Braak I+^ vs. A+T-	0.8 (0.7, 0.91)	0.22	0.68 (0.39, 0.82)	0.83 (0.61, 1)	0.7 (0.51, 0.81)

Abbreviations: Thr., Threshold identified using Youden's index; Sens., Sensitivity; Spec., Specificity; Acc., Accuracy; CInt, confidence interval.

**Table 5:** Accuracy analysis of blood biomarkers in WRAP longitudinal study.

Analyte	Comparison	AUC (95% CInt)	Thr.	Sens. (95% CInt)	Spec. (95% CInt)	Acc. (95% CInt)
pTau-181 (pg/mL)	T^Braak V+^ vs. T- and T^Braak I–IV^	0.85 (0.7, 1)	3.72	0.82 (0.77, 0.86)	0.89 (0.85, 0.92)	0.88 (0.84, 0.91)
ALZpath pTau-217 (pg/mL)	T^Braak V+^ vs. T- and T^Braak I–IV^	0.95 (0.89, 1)	1.18	0.82 (0.77, 0.86)	0.97 (0.95, 0.99)	0.96 (0.94, 0.98)
pTau-231 (pg/mL)	T^Braak V+^ vs. T- and T^Braak I–IV^	0.85 (0.71, 0.99)	15.55	0.82 (0.77, 0.86)	0.8 (0.76, 0.84)	0.81 (0.76, 0.85)
pTau-181 (pg/mL)	T^Braak I+^ vs. T-	0.74 (0.66, 0.81)	2.87	0.59 (0.53, 0.64)	0.72 (0.66, 0.76)	0.7 (0.64, 0.74)
ALZpath pTau-217 (pg/mL)	T^Braak I+^ vs. T-	0.78 (0.71, 0.85)	0.62	0.57 (0.52, 0.63)	0.84 (0.79, 0.87)	0.79 (0.74, 0.83)
pTau-231 (pg/mL)	T^Braak I+^ vs. T-	0.72 (0.64, 0.8)	13.25	0.55 (0.5, 0.61)	0.67 (0.61, 0.72)	0.65 (0.59, 0.7)
pTau-181 (pg/mL)	A+T^Braak V+^ vs. A+T- or A+T^Braak I–IV^	0.94 (0.88, 1)	3.96	0.89 (0.8, 0.94)	0.79 (0.68, 0.86)	0.9 (0.81, 0.95)
ALZpath pTau-217 (pg/mL)	A+T^Braak V+^ vs. A+T- or A+T^Braak I–IV^	0.97 (0.94, 1)	1.18	1 (0.95, 1.01)	0.87 (0.78, 0.93)	0.96 (0.89, 0.99)
pTau-231 (pg/mL)	A+T^Braak V+^ vs. A+T- or A+T^Braak I–IV^	0.83 (0.68, 0.97)	16.45	0.78 (0.67, 0.85)	0.66 (0.55, 0.76)	0.84 (0.74, 0.9)
pTau-181 (pg/mL)	A+T^Braak I+^ vs. A+T-	0.67 (0.55, 0.8)	3.72	0.5 (0.39, 0.61)	0.8 (0.7, 0.87)	0.82 (0.73, 0.89)
ALZpath pTau-217 (pg/mL)	A+T^Braak I+^ vs. A+T-	0.72 (0.6, 0.84)	0.78	0.67 (0.55, 0.76)	0.66 (0.55, 0.76)	0.84 (0.74, 0.9)
pTau-231 (pg/mL)	A+T^Braak I+^ vs. A+T-	0.61 (0.48, 0.74)	14.85	0.56 (0.44, 0.65)	0.55 (0.44, 0.65)	0.73 (0.63, 0.82)

Abbreviations: Thr., Threshold identified using Youden's index; Sens., Sensitivity; Spec., Specificity; Acc., Accuracy; CInt, confidence interval.

Next, we set out to investigate these findings in different subpopulations in the TRIAD cohort. First, we analysed the ROC characteristic of an age matched cohort where only the participants older than 65 years were included (A-T-: *n* = 110, mean age = 72, standard deviation (SD) age = 4.8; A+T-: *n* = 22, mean age = 73, SD age = 5.1; A+T^Braak I–IV^: *n* = 35, mean age = 74, SD age = 5.0; A+T^Braak V+^: *n* = 35, mean age = 72, SD age = 4.0). Confirmatory, we found that pTau-217+^Janssen^, and ALZpath pTau-217 identified T^Braak V+^ individuals with an AUC >0.9 in all participants and in A+ preselected participants (Supplementary Figure S4, the results of the statistical comparisons are provided in Supplementary Table S4). Furthermore, we analysed the ROC characteristics in individuals with mild cognitive impairment and mild dementia (CDR score ≤1 and MMSE >21) to investigate which tau analytes could be used to determine eligibility for Aβ-targeting therapies (A-T-: *n* = 18, mean age = 70, SD age = 10.6; A+T-: *n* = 9, mean age = 72, SD age = 6.6; A+T^Braak I–IV^: *n* = 18, mean age = 71, SD age = 9.5; A+T^Braak V+^: *n* = 34, mean age = 71, SD age = 6.1). Again, our analyses showed that pTau-217+^Janssen^, and ALZpath pTau-217 identified T^Braak V+^ individuals with an AUC >0.9 in all participants and in A+ preselected participants (Supplementary Figure S5, the results of the statistical comparisons are provided in Supplementary Table S5). Next, we tested the ROC characteristics to identify Braak-dependent tau accumulation in cognitively impaired (CI) and unimpaired (CU) participants. Therefore, we set out to identify CI A+T^Braak V+^ (*n* = 41) vs. CI A+T- and A+T^Braak I-IV^ (*n* = 41) participants. In this analysis, pTau-217+^Janssen^ (AUC = 0.98), ALZpath pTau-217 (AUC = 0.91), and NTA-tau (AUC = 0.93) showed the best performance. Next, we probed the ROC characteristics of the tau analytes to separate CU A+ with any tau accumulation (*n* = 16) from those without tau accumulation (*n* = 14). Again, pTau-217+^Janssen^ (AUC = 0.94), and ALZpath pTau-217 (AUC = 0.87) achieved the best separation (Supplementary Figure S6, the results of the statistical comparisons are provided in Supplementary Table S3). Thus, we concluded that pTau-217 identifies individuals with tau accumulation in later Braak stages in different AD relevant subpopulations.

Next, we performed a *post-hoc* power analysis separately for TRIAD and WRAP. Given the respective sample sizes and a 0.05 significance level, our study had a power of 1.0 to differentiate T^Braak V+^ in all or A+ individuals with an AUC of 0.80 which corresponded to the lowest AUC that we observed in our analyses across all tau derivates. Thus, our study was adequately powered to identify T^Braak V+^ individuals with the respective blood biomarkers.

### Braak V+ tau PET positivity can be estimated by adapted cut-offs

**Fig. 3:**
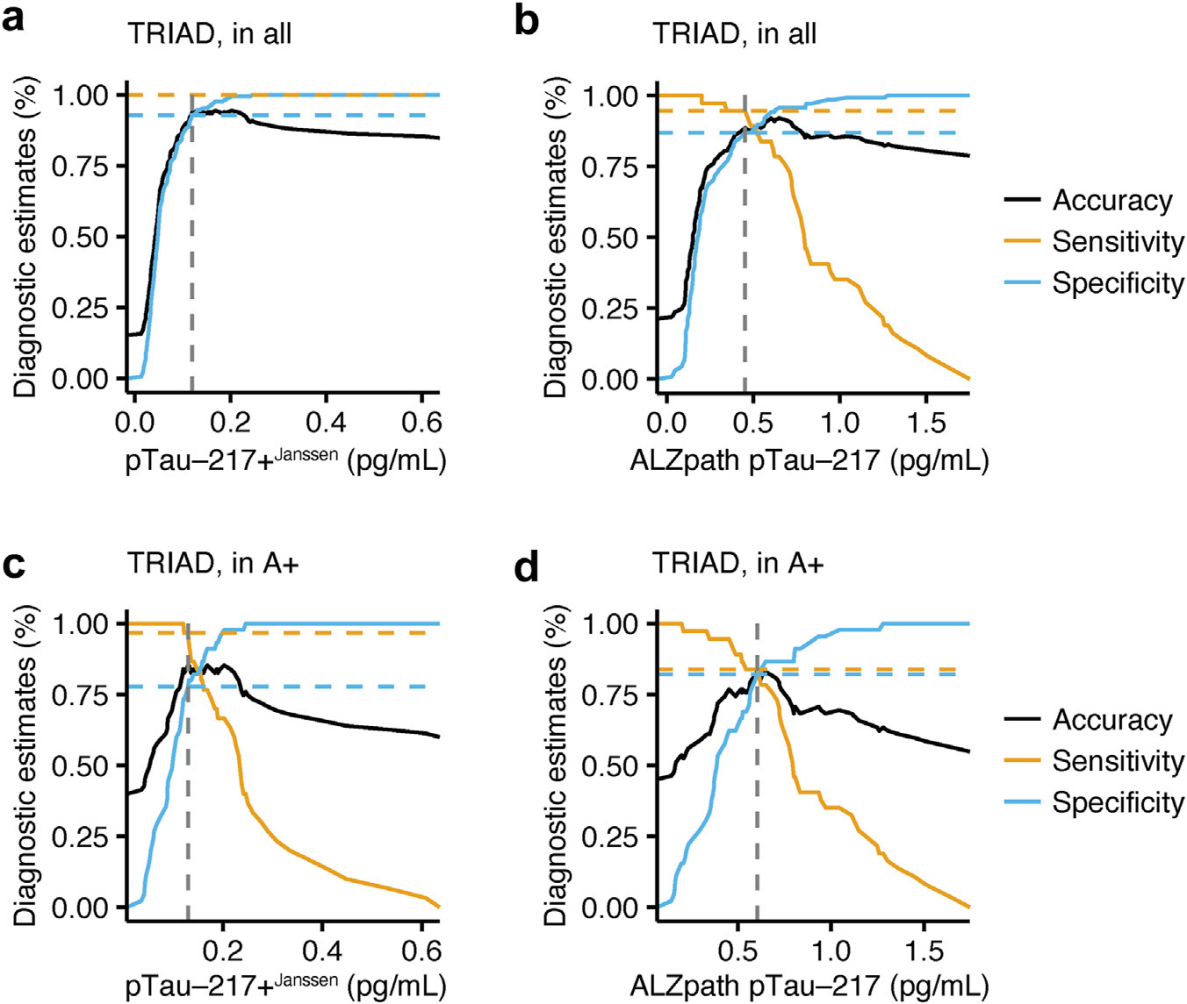
**pTau-217 identifies individuals with Braak V**^**+**^**tau accumulation in TRIAD**. (a and b) Accuracy, sensitivity, and specificity of pTau-217+^Janssen^ in TRIAD (a), and ALZpath pTau-217 in TRIAD (b) to detect T^Braak V+^ individuals without preselection. (c and d) Accuracy, sensitivity, and specificity of pTau-217+^Janssen^ in TRIAD (c), ALZpath pTau-217 in TRIAD (d) to detect T^Braak V+^ individuals in Aβ+ participants. Cut-off according to Youden-index are shown as dashed lines. pTau, phospho-tau.

Next, we determined different cut-offs for all participants without preselection and Aβ+ individuals to separate (A) T^Braak I+^ individuals from T- participants, and (B) T^Braak V+^ participants from all other participants, including those with no tau and those with tau accumulation in lower Braak stages (T^Braak I–IV^). Therefore, we calculated absolute thresholds for optimal separation using Youden's index from our previous ROC analyses for pTau-181, pTau-217+^Janssen^, ALZpath pTau-217 and NTA-tau (all thresholds, sensitivity, specificity, and accuracy analyses are shown in Table 4). Across all biomarkers, an increase of the threshold led to a separation of T^Braak V+^ individuals from T^Braak I–IV^ and T- individuals with or without preselection by brain amyloidosis. We focused on our two pTau-217 assays since they consistently showed the highest accuracy to differentiate tau PET-positive participants from others without and with preselection of Aβ+ participants. In participants without preselection, 0.12 pg/mL pTau-217+^Janssen^ (Fig. 3a; sensitivity = 1, specificity = 0.93, accuracy = 0.94), and 0.45 pg/mL ALZpath pTau-217 (Fig. 3b; sensitivity = 0.95, specificity = 0.89, accuracy = 0.90) separated T^Braak V+^ individuals in the TRIAD cohort (in comparison to 0.09 pg/mL pTau-217+^Janssen^ and 0.32 pg/mL ALZpath pTau-217 to identify T^Braak I+^ individuals). We repeated the analyses after preselection of Aβ+ participants. Here, pTau-217+^Janssen^ blood levels >0.13 pg/mL (Fig. 3c; sensitivity = 0.97, specificity = 0.82, accuracy = 0.88), and ALZpath pTau-217 blood levels >0.6 pg/mL (Fig. 3d; sensitivity = 0.84, specificity = 0.84, accuracy = 0.84) identified A+T^Braak V+^ tau PET-positive participants in the TRIAD cohort (in comparison to 0.09 pg/mL pTau-217+^Janssen^ and 0.39 pg/mL ALZpath pTau-217 to identify A+T^Braak I+^ tau PET-positive participants).

**Fig. 4:**
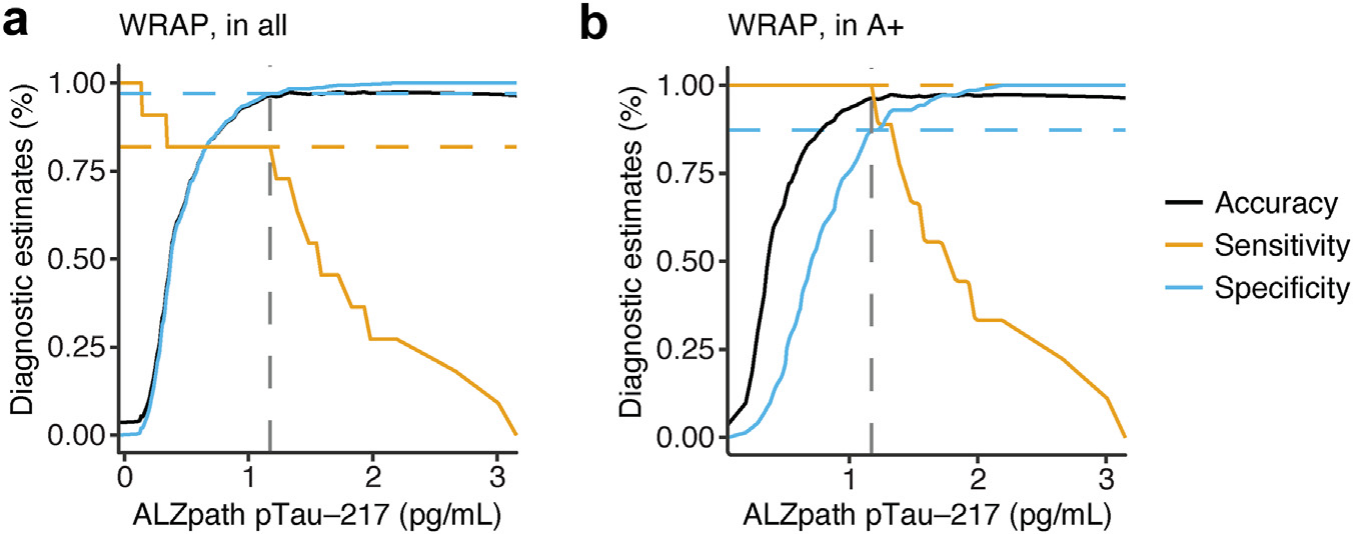
**pTau-217 identifies individuals with Braak V**^**+**^**tau accumulation in WRAP**. (a and b) Accuracy, sensitivity, and specificity of ALZpath pTau-217 in WRAP to detect T^Braak V+^ individuals without preselection (a) and in Aβ+ participants (b). Cut-off according to Youden-index are shown as dashed lines. pTau, phospho-tau.

Using a similar procedure in the independent WRAP study cohort, ALZpath pTau-217 identified T^Braak V+^ participants with a 1.18 pg/mL cut-off (Fig. 4a; sensitivity = 0.82, specificity = 0.97, accuracy = 0.96). Furthermore, ALZpath pTau-217 identified A+T^Braak V+^ tau PET-positive participants with a 1.18 pg/mL cut-off (Fig. 4b; all statistical results for the WRAP study cohort are shown in Table 5; sensitivity = 1.0, specificity = 0.87, accuracy = 0.96) underlining that blood levels of pTau-217 can be used to identify individuals with late-stage cortical tau accumulation.

Last, we aimed to analyse whether the prediction of T^Braak V+^ tau accumulation benefits from combining pTau-217 with pTau-231 or pTau-181. Therefore, we performed an agreement analysis of the PET-based with a blood-based T^Braak V+^ classification using our respective cut-offs. Across both cohorts, we found a high agreement between the PET-based and ALZpath pTau-217-based identification of T^Braak V+^ in all or in A+ preselected participants with a Cohen's Kappa coefficient ≥0.6. However, combining ALZpath pTau-217 with the other biomarker consistently reduced the agreement between the PET-based and blood-based identification of T^Braak V+^ in the TRIAD and WRAP cohorts. Similarly, pTau-217+^Janssen^ that was only available in the TRIAD cohort showed a high agreement with the PET-based identification of T^Braak V+^ participants that was decreased by combining pTau-181 or pTau-231 (Supplementary Table S6).

## Discussion

AD stage has implication on the outcomes of disease modifying treatments.^44^, ^45^, ^46^ This study assessed the ability of plasma biomarkers to identify individuals within a late-stage tau accumulation, designated as a PET-based Braak stage of V+. We found that plasma pTau-217 could reliably detect late-stage tau accumulation in amyloid-PET-positive individuals. Moreover, we identified cut-offs that were able to identify late-stage tau accumulation in an independent cohort. These results support the construct that plasma pTau concentrations is useful in estimating biological severity in AD and for determining eligibility for clinical trials by excluding individuals with high tau burden.

The recent TRAILBLAZER-ALZ2 study observed that patients with more advanced tau pathology experienced lower clinical benefit in response to amyloid-PET reduction. In light of these findings, there may be a need to determine severity of a patient's biological AD in order to forecast someone's response to treatment. In particular, there is a clinical need to identify individuals with tau accumulation in later Braak stages who are not eligible for Aβ-targeting therapies. Thus, plasma pTau-217 provides useful information in this regard by estimating tau-PET severity. Notably, this was consistent in two separate cohorts recruited in a specialized tertiary care memory clinic (TRIAD) and longitudinal observational study (WRAP) with different demographics supporting the generalizability of our results. Future studies are needed to determine whether a combination of plasma biomarkers (i.e., a plasma biomarker panel^47^) will provide additional information to continuous plasma pTau-217 concentrations.

Because of the close association between tau-PET and cognitive decline,^48^^,^^49^ elevated thresholds for plasma p-tau may suggest patients are at higher risk of future cognitive decline. In fact, a recent study that divided individuals based on plasma pTau-217 quartiles provided evidence that elevated plasma pTau-217 concentrations were associated with a higher risk of cognitive decline over 6 years.^50^ Since we did not find significant associations between pTau-217 and hippocampal volume when separately analysing individuals with high and low tau burden, our data suggests that pTau-217 rather specifically reflects AD pathophysiology than broadly neurodegeneration. Therefore, our study provides further support to the notion that using higher thresholds for plasma pTau-217 can be used to estimate risk of AD-related cognitive decline.

From a diagnostic standpoint, there may also be a use-case for different plasma pTau-217 thresholds. A recent study used a two-step workflow where patients with MCI were characterised as low risk/intermediate risk/high risk for elevated brain amyloid-PET based on plasma pTau-217 concentrations.^51^ The use of a three-range plasma pTau-217 approach (as opposed to a normal/abnormal cut-point) was associated with a lower number of cognitively impaired patients who would require confirmatory biomarker testing.^51^ Another study showed that pTau-217 as stand-alone test can reliably identify A+ individuals including individuals with high tau burden who would require confirmatory tau-PET.^52^ Our study contributes to this framework by highlighting that elevated (i.e., highly abnormal) concentrations of plasma pTau-217 are not only highly likely to be associated with elevated amyloid-PET but also associated with advanced tau accumulation. Along these lines, we additionally show that pTau-217 can identify individuals with MCI or mild AD who would be disqualified from Aβ-targeting therapies due to the high tau burden. Furthermore, our data suggests that pTau-217 alone shows a higher agreement with the PET-based classification of tau accumulation in later Braak stages than combining pTau-217 with other tau analytes. Thus, blood pTau-217 in combination with the clinical phenotype might suffice for AD diagnosis, disease staging and eligibility testing for Aβ-targeting therapies. However, further studies in diverse populations and patient groups are required to investigate pTau-217 as stand-alone test for estimating AD severity.

While many studies have focused on identifying abnormal amyloid-PET using plasma biomarkers, a limitation is that plasma pTau-217 alone cannot determine whether AD is driving a patient's clinical phenotype, or whether the abnormal Aβ is incidental. Another advantage of using higher thresholds to detect advanced tau accumulation is that it increases the likelihood that the clinical phenotype is driven by AD. Despite these potential advantages, prospective studies evaluating how plasma biomarkers influence diagnosis and care of patients with neurodegenerative diseases are needed (i.e., similar to the IDEAS study^53^).

Our study has limitations. The first limitation is that both the TRIAD and WRAP cohorts constitute self-selected individuals who are interested in participating in aging and dementia research. Both samples are also highly educated and feature a low proportion of non-white individuals. Therefore, replication of the present results in more representative populations and different patient care settings is needed. Another limitation of the present study is that both the TRIAD and WRAP studies are single-centre studies where plasma biomarker collection and analysis protocols are more tightly controlled than can be reasonably achieved in large multicentre studies. Furthermore, our findings need to be replicated in multi-ethnic cohorts to incorporate our findings into community-based diagnostic procedures. Furthermore, although we included possible confounders in our analyses, studies with larger sample sizes should determine separate cut-offs for age groups, sexes and genetic risk factors which could further guide AD diagnosis and staging. A final limitation of our study is that plasma biomarkers were measured for each assay all at once (which is standard practice in cohort studies). Therefore, before these assays and cutoff points can be used in clinical practice, they need to be validated in a prospective manner.

In summary, this study highlights that pTau-217 blood concentration qualifies as biomarker to identify individuals with tau accumulation in Braak stages V and VI which has the potential to guide patient stratification for treatments, clinical trials, and patient counselling.

## Contributors

MSW, JT, EMJ, RW, REL, SCJ, and PRN created the concept and design of the study.

Data acquisition and analysis was performed by MSW, JT, EMJ, RW, REL, NR, ALB, NJA, CT, JLR, ACM, SS, YTW, JFA, SAH, TJB, FZL, RH, GTB, HCK, AJ, EK, GM, MAF, TAP, SG, HZ, KB, SCJ, PV, JK and PRN contributed to the sample selection/and or interpretation of the data. JT, NJA and ALB verified the underlying data. MSW, JT, EMJ, RW, REL, SCJ, and PRN drafted the manuscript and all authors revised. All authors read and approved the final manuscript.

## Data sharing statement

This study includes no data deposited in external repositories. Data from TRIAD that support the findings of the study are available from the corresponding authors upon reasonable request. Requests for WRAP data should be submitted to the WRAP executive committee via this website: https://wrap.wisc.edu/data-requests-2/.

## Declaration of interests

H.Z. has served at scientific advisory boards and/or as a consultant for Abbvie, Acumen, Alector, Alzinova, ALZPath, Annexon, Apellis, Artery Therapeutics, AZTherapies, Cognito Therapeutics, CogRx, Denali, Eisai, Merry Life, Nervgen, Novo Nordisk, Optoceutics, Passage Bio, Pinteon Therapeutics, Prothena, Red Abbey Labs, reMYND, Roche, Samumed, Siemens Healthineers, Triplet Therapeutics, and Wave, has given lectures in symposia sponsored by Alzecure, Biogen, Cellectricon, Fujirebio, Lilly, and Roche, and is a co-founder of Brain Biomarker Solutions in Gothenburg AB (BBS), which is a part of the GU Ventures Incubator Program (outside submitted work). K.B. has served as a consultant and at advisory boards for Acumen, ALZPath, BioArctic, Biogen, Eisai, Julius Clinical, Lilly, Novartis, Ono Pharma, Prothena, Roche Diagnostics, and Siemens Healthineers; has served at data monitoring committees for Julius Clinical and Novartis; has given lectures, produced educational materials and participated in educational programs for Biogen, Eisai and Roche Diagnostics; and is a co-founder of Brain Biomarker Solutions in Gothenburg AB (BBS), which is a part of the GU Ventures Incubator Program, outside the work presented in this paper. H.C.K and G.T.B receive salary and stock from Janssen R&D. All other authors declare no conflicts of interest. E.M.J. served on DSMB for the NIA study K01 AG073587. S.G. received consulting fees as a member of the scientific advisory boards in Abbvie, Alzheon, AmyriAD, Eisai, Enigma/Meilleur, Lilly, Okutsa, Novo Nordisk, TauRx, honoraria for educational videos from Lundbeck, reimbursement for AD/PD 2024 travel expenses by TauRx. S.G. is board member at the Sharon and Robert Francis Foundation, Toronto, Canada, and the Canadian Conference on Dementia (CCD). S.C.J. received payment for consulting Enigma Biomedical and consulted ALZpath without payment. T.J.B. received funding from NIH/NIA (R01AG080766), personal honoraria from the NIH, Intermountain Healthcare, reimbursements for travel by University College London, Alzheimer's Association, and NIH. J.T. served as a Consultant for the Neurotorium Educational platform and for Alzheon. P.V. received consulting fees from Eisai, Novo Nordisk, honoraria from Astra, support for travelling by Lilly, and participated on DMSB for the IntelGenxCorp. Y.T.W. has written an educational article for the Neurotorium Educational platform. P.R.N. received consulting fees from Novo Nordisk, Eisai, and honoraria for an academic talk from Novo Nordisk.
