# Supplementary material for: Identification of late-stage tau accumulation using plasma phospho-tau217
Source: eBioMedicine. 2024 Nov 4;109:105413. doi: 10.1016/j.ebiom.2024.105413 (PMC11570195; doi:10.1016/j.ebiom.2024.105413)
Supplement: Supplementary [file mmc1.docx]

**Supplementary data**

**Supplementary Table S1. Patient characteristics separated by sex.**

|  |  | Sex | A-T- | A+T- | A+T^Braak I-IV^ | A+T^Braak V+^ | A-T^Braak I-IV^ | A-T^Braak V+^ |
| --- | --- | --- | --- | --- | --- | --- | --- | --- |
| TRIAD | N | M | 66 | 12 | 13 | 25 | 0 | 0 |
| TRIAD |  | F | 100 | 14 | 26 | 33 | 0 | 0 |
| TRIAD | Diagnosis, Cognitively unimpaired, N (%) | M | 51 (77.3) | 7 (58.3) | 4 (30.8) | 1 (4.0) | NA | NA |
| TRIAD |  | F | 86 (86.0) | 9 (64.3) | 16 (61.5) | 0 (0.0) | NA | NA |
| TRIAD | Diagnosis, Mild cognitive impairment, N (%) | M | 15 (22.7) | 5 (41.7) | 6 (46.2) | 8 (32.0) | NA | NA |
| TRIAD |  | F | 14 (14.0) | 5 (35.7) | 9 (34.6) | 12 (36.4) | NA | NA |
| TRIAD | Diagnosis, Dementia, N (%) | M | 0 (0.0) | 0 (0.0) | 3 (23.1) | 16 (64.0) | NA | NA |
| TRIAD |  | F | 0 (0.0) | 0 (0.0) | 1 (3.8) | 21 (63.6) | NA | NA |
| TRIAD | *APOE* ε4 carriership, N (%) | M | 22 (33.3) | 5 (41.7) | 6 (46.2) | 17 (68.0) | NA | NA |
| TRIAD |  | F | 22 (22.0) | 0 (0.0) | 12 (46.2) | 27 (81.8) | NA | NA |
| TRIAD | Age (years), mean (SD) | M | 59 (19.3) | 72.7 (5.8) | 72 (11.1) | 64.8 (10.0) | NA | NA |
| TRIAD |  | F | 59.8 (20.3) | 71.2 (8.0) | 72.4 (8.4) | 66.7 (7.9) | NA | NA |
| TRIAD | Mini-Mental State Exam, mean (SD) | M | 28.3 (2.9) | 28.4 (2.2) | 27.2 (3.5) | 22.2 (5.9) | NA | NA |
| TRIAD |  | F | 29.2 (1.3) | 26.9 (6.0) | 28.7 (1.4) | 23.2 (5.6) | NA | NA |
| TRIAD | Educational years, mean (SD) | M | 14.8 (3.9) | 16 (2.6) | 15.5 (3.8) | 15.1 (3.3) | NA | NA |
| TRIAD |  | F | 15.5 (3.4) | 12.9 (2.8) | 14.6 (3.5) | 14.5 (3.7) | NA | NA |
| TRIAD | Total tau SUVR, mean (SD) | M | 0.9 (0.1) | 0.9 (0.1) | 1 (0.1) | 1.9 (0.6) | NA | NA |
| TRIAD |  | F | 0.9 (0.1) | 0.9 (0.1) | 1 (0.1) | 2.1 (1.0) | NA | NA |
| TRIAD | Total amyloid DVR, mean (SD) | M | 1.3 (0.1) | 1.8 (0.4) | 2.2 (0.3) | 2.3 (0.4) | NA | NA |
| TRIAD |  | F | 1.2 (0.1) | 1.8 (0.3) | 2.1 (0.4) | 2.5 (0.5) | NA | NA |
| TRIAD | Hippocampal volume, mean, cm^3^, (SD) | M | 3.8 (0.6) | 3.4 (0.5) | 3.5 (0.5) | 3.1 (0.6) | NA | NA |
| TRIAD |  | F | 3.5 (0.4) | 3.4 (0.3) | 3.3 (0.4) | 2.9 (0.4) | NA | NA |
| WRAP | N | M | 70 | 18 | 9 | 1 | 3 | 0 |
| WRAP |  | F | 139 | 26 | 18 | 8 | 12 | 2 |
| WRAP | Diagnosis, Cognitively unimpaired, N (%) | M | 70 (100.0) | 16 (88.9) | 9 (100.0) | 1 (100.0) | 3 (100.0) | NA |
| WRAP |  | F | 138 (99.3) | 26 (100.0) | 15 (83.3) | 4 (50.0) | 12 (100.0) | 2 (100.0) |
| WRAP | Diagnosis, Mild cognitive impairment, N (%) | M | 0 (0.0) | 2 (11.1) | 0 (0.0) | 0 (0.0) | 0 (0.0) | NA |
| WRAP |  | F | 1 (0.7) | 0 (0.0) | 3 (16.7) | 3 (37.5) | 0 (0.0) | 0 (0.0) |
| WRAP | Diagnosis, Dementia, N (%) | M | 0 (0.0) | 0 (0.0) | 0 (0.0) | 0 (0.0) | 0 (0.0) | NA |
| WRAP |  | F | 0 (0.0) | 0 (0.0) | 0 (0.0) | 1 (12.5) | 0 (0.0) | 0 (0.0) |
| WRAP | *APOE* ε4 carriership, N (%) | M | 20 (28.6) | 12 (66.7) | 9 (100.0) | 0 (0.0) | 1 (33.3) | NA |
| WRAP |  | F | 42 (30.2) | 14 (54.8) | 11 (61.1) | 6 (75.0) | 5 (41.7) | 1 (50.0) |
| WRAP | Age (years), mean (SD) | M | 66.3 (6.7) | 69.4 (5.4) | 69.5 (5.8) | 65.6 (0.0) | 66.9 (10.2) | NA |
| WRAP |  | F | 65.4 (6.7) | 68.8 (5.4) | 68.9 (6.7) | 69.4 (4.2) | 68.6 (6.6) | 57.4 (2.9) |
| WRAP | Mini-Mental State Exam, mean (SD) | M | 29.4 (0.7) | 28.8 (1.4) | 28.9 (1.5) | 28.0 (0.0) | 30.0 (0.0) | NA |
| WRAP |  | F | 29.5 (0.7) | 29.4 (0.8) | 28.9 (1.2) | 26.9 (4.5) | 29.4 (0.7) | 29.0 (0.0) |
| WRAP | Educational years, mean (SD) | M | 17.01 (2.8) | 16.9 (2.7) | 17.8 (2.7) | 16.0 (0.0) | 15.3 (3.1) | NA |
| WRAP |  | F | 15.8 (2.6) | 16.2 (2.2) | 15.7 (2.1) | 15.0 (2.8) | 15.9 (2.8) | 18.0 (0.0) |
| WRAP | Total tau SUVR, mean (SD) | M | 1.0 (0.1) | 0.8 (0.1) | 1.1 (0.1) | 1.5 (0.0) | 1.1 (0.1) | NA |
| WRAP |  | F | 1.1 (0.1) | 1.1 (0.1) | 1.1 (0.1) | 1.9 (0.4) | 1.1 (0.1) | 1.4 (0.01) |
| WRAP | Total amyloid DVR, mean (SD) | M | 1.1 (0.1) | 1.4 (0.2) | 1.5 (0.2) | 1.9 (0.0) | 1.1 (0.04) | NA |
| WRAP |  | F | 1.1 (0.1) | 1.4 (0.2) | 1.6 (0.2) | 1.8 (0.2) | 1.1 (0.04) | 1.1 (0.02) |
| WRAP | Hippocampal volume, mean, cm^3^, (SD) | M | 3.9 (0.5) | 4.1 (0.4) | 3.8 (0.4) | 3.4 (0.0) | 3.9 (0.2) | NA |
| WRAP |  | F | 3.8 (0.4) | 3.7 (0.3) | 3.5 (0.4) | 3.5 (0.6) | 3.5 (0.4) | 3.8 (0.3) |

Abbreviations: M = male, F = female

**Supplementary Table S2. Correlation of tau analytes with hippocampal volume.**

|  | **Group** | ***P*-value** | **R (95% CI)** |
| --- | --- | --- | --- |
| pTau-181 | A-T- | 0.48 | -0.055 (-0.21, 0.099) |
| pTau-181 | A+T^V+^ | 0.15 | -0.20 (-0.44, 0.072) |
| pTau-181 | A+T^Braak I-IV^ | 0.15 | -0.24 (-0.52, 0.092) |
| pTau-181 | A+T- | 0.36 | -0.20 (-0.56, 0.23) |
| pTau-217+^Janssen^ | A-T- | 0.13 | -0.13 (-0.29, 0.039) |
| pTau-217+^Janssen^ | A+T^V+^ | 0.39 | 0.14 (-0.19, 0.43) |
| pTau-217+^Janssen^ | A+T^Braak I-IV^ | 0.81 | -0.045 (-0.39, 0.31) |
| pTau-217+^Janssen^ | A+T- | 0.34 | -0.20 (-0.56, 0.22) |
| pTau-231 | A-T- | 0.67 | 0.034 (-0.12, 0.19) |
| pTau-231 | A+T^V+^ | 0.033 | -0.28 (-0.50, -0.024) |
| pTau-231 | A+T^Braak I-IV^ | 0.71 | -0.063 (-0.38, 0.27) |
| pTau-231 | A+T- | 0.51 | -0.14 (-0.52, 0.28) |
| Total tau | A-T- | 0.012 | 0.23 (0.052, 0.39) |
| Total tau | A+T^V+^ | 0.80 | -0.041 (-0.35, 0.27) |
| Total tau | A+T^Braak I-IV^ | 0.93 | 0.017 (-0.35, 0.38) |
| Total tau | A+T- | 0.53 | -0.17 (-0.61, 0.36) |
| NTA tau | A-T- | 0.43 | 0.067 (-0.097, 0.23) |
| NTA tau | A+T^V+^ | 0.75 | -0.048 (-0.33, 0.24) |
| NTA tau | A+T^Braak I-IV^ | 0.62 | -0.083 (-0.39, 0.24) |
| NTA tau | A+T- | 0.20 | -0.27 (-0.61, 0.15) |
| ALZpath pTau-217 | A-T- | 0.055 | -0.18 (-0.36, 0.0042) |
| ALZpath pTau-217 | A+T^V+^ | 0.16 | -0.21 (-0.47, 0.081) |
| ALZpath pTau-217 | A+T^Braak I-IV^ | 0.31 | 0.18 (-0.17, 0.50) |
| ALZpath pTau-217 | A+T- | 0.85 | 0.041 (-0.38, 0.45) |

Abbreviations: pTau = phospho-tau, NTA tau = N-terminal tau, CI = confidence interval

**Supplementary Table S3. ROC analyses of all participants in TRIAD and WRAP.**

|  | Cohort | Comparison | pTau-181 | ALZpath pTau-217 | pTau-231 | NTA-tau | pTau-217+^Janssen^ |
| --- | --- | --- | --- | --- | --- | --- | --- |
| pTau-217+^Janssen^ | TRIAD | AD vs. non-AD | 0.89 | 1.0 | 0.20 | 0.59 | NA |
| pTau-217+^Janssen^ | TRIAD | A+T- vs. A-T- | 0.092 | 1.0 | 0.14 | 0.015 | NA |
| pTau-217+^Janssen^ | TRIAD | A+T+ vs. A-T- | 0.013 | 1.0 | 0.0052 | 0.0082 | NA |
| pTau-217+^Janssen^ | TRIAD | CI A+T^Braak V+^ vs. CI A+ rest | 0.091 | 0.59 | 0.065 | 0.67 | NA |
| pTau-217+^Janssen^ | TRIAD | CU A+T^Braak I+^ vs. CU A+T- | 0.64 | 1.0 | 0.44 | 0.23 | NA |
| pTau-217+^Janssen^ | TRIAD | T^Braak I+^ vs. T- | 0.87 | 1.0 | 0.33 | 0.30 | NA |
| pTau-217+^Janssen^ | TRIAD | T^Braak V+^ vs. rest | 0.014 | 0.52 | 0.0071 | 0.021 | NA |
| pTau-217+^Janssen^ | TRIAD | A+T^Braak I+^ vs. A+T- | 0.42 | 0.35 | 0.12 | 0.11 | NA |
| pTau-217+^Janssen^ | TRIAD | A+T^Braak V+^ vs. A+ rest | 0.27 | 1.0 | 0.092 | 1.0 | NA |
| ALZpath pTau-217 | TRIAD | AD vs. non-AD | 1.0 | NA | 1.0 | 1.0 | 1.0 |
| ALZpath pTau-217 | TRIAD | A+T- vs. A-T- | 0.022 | NA | 0.031 | 0.0035 | 1.0. |
| ALZpath pTau-217 | TRIAD | A+T+ vs. A-T- | 0.29 | NA | 0.16 | 0.22 | 10 |
| ALZpath pTau-217 | TRIAD | CI A+T^Braak V+^ vs. CI A+ rest | 1.0 | NA | 1.0 | 1.0 | 0.59 |
| ALZpath pTau-217 | TRIAD | CU A+T^Braak I+^ vs. CU A+T- | 1.0 | NA | 1.0 | 0.92 | 1.0. |
| ALZpath pTau-217 | TRIAD | T^Braak I+^ vs. T- | 1.0 | NA | 0.52 | 0.45 | 10 |
| ALZpath pTau-217 | TRIAD | T^Braak V+^ vs. rest | 1.0 | NA | 0.99 | 1.0 | 0.52 |
| ALZpath pTau-217 | TRIAD | A+T^Braak I+^ vs. A+T- | 1.0 | NA | 1.0 | 1.0 | 0.35 |
| ALZpath pTau-217 | TRIAD | A+T^Braak V+^ vs. A+ rest | 1.0 | NA | 1.0 | 1.0 | 1.0 |
| ALZpath pTau-217 | WRAP | T^Braak I+^ vs. T- | 0.17 | NA | 0.16 | NA | NA |
| ALZpath pTau-217 | WRAP | T^Braak V+^ vs. rest | 0.26 | NA | 0.23 | NA | NA |
| ALZpath pTau-217 | WRAP | A+T^Braak I+^ vs. A+T- | 0.29 | NA | 0.16 | NA | NA |
| ALZpath pTau-217 | WRAP | A+T^Braak V+^ vs. A+ rest | 0.26 | NA | 0.16 | NA | NA |

The *P*-values of DeLong’s comparison between the indicated ROC-analyses are shown. Abbreviations: rest = T^Braak I-IV^ and T-, pTau = phospho-tau, CI = cognitively impaired, CU = cognitively unimpaired

**Supplementary Table S4. ROC analyses of participants older than 65 years in TRIAD.**

|  | Comparison | pTau-181 | pTau-217+^Janssen^ | ALZpath pTau-217 | pTau-231 | NTA-tau |
| --- | --- | --- | --- | --- | --- | --- |
| pTau-217+^Janssen^ | AD vs. non-AD | 1.0 | NA | 1.0 | 0.11 | 0.44 |
| pTau-217+^Janssen^ | T^Braak I+^ vs. T- | 1.0 | NA | 1.0 | 0.28 | 0.55 |
| pTau-217+^Janssen^ | T^Braak V+^ vs. rest | 0.066 | NA | 0.94 | 0.032 | 0.044 |
| pTau-217+^Janssen^ | A+T^Braak I+^ vs. A+T- | 0.33 | NA | 0.16 | 0.061 | 0.12 |
| pTau-217+^Janssen^ | A+T^Braak V+^ vs. A+ rest | 0.13 | NA | 0.47 | 0.027 | 0.21 |
| ALZpath pTau-217 | AD vs. non-AD | 1.0 | 1.0 | NA | 0.84 | 1.0 |
| ALZpath pTau-217 | T^Braak I+^ vs. T- | 1.0 | 1.0 | NA | 0.73 | 1.0 |
| ALZpath pTau-217 | T^Braak V+^ vs. rest | 1.0 | 0.94 | NA | 1.0 | 1.0 |
| ALZpath pTau-217 | A+T^Braak I+^ vs. A+T- | 1.0 | 0.16 | NA | 1.0 | 1.0 |
| ALZpath pTau-217 | A+T^Braak V+^ vs. A+ rest | 1.0 | 0.47 | NA | 1.0 | 1.0 |

The *P*-values of DeLong’s comparison between the indicated ROC-analyses are shown. Abbreviations: rest = T^Braak I-IV^ and T-, pTau = phospho-tau

**Supplementary Table S5. ROC analyses of MCI and mild AD participants in TRIAD.**

|  | Comparison | pTau-181 | pTau-217+^Janssen^ | ALZpath pTau-217 | pTau-231 | NTA-tau |
| --- | --- | --- | --- | --- | --- | --- |
| pTau-217+^Janssen^ | AD vs. non-AD | 1.0 | NA | 1.0 | 1.0 | 1.0 |
| pTau-217+^Janssen^ | T^Braak I+^ vs. T- | 1.0 | NA | 1.0 | 0.79 | 0.40 |
| pTau-217+^Janssen^ | T^Braak V+^ vs. rest | 0.19 | NA | 0.69 | 0.59 | 1.0 |
| pTau-217+^Janssen^ | A+T^Braak I+^ vs. A+T- | 1.0 | NA | 1.0 | 1.0 | 1.0 |
| pTau-217+^Janssen^ | A+T^Braak V+^ vs. A+ rest | 0.27 | NA | 1.0 | 0.52 | 1.0 |
| ALZpath pTau-217 | AD vs. non-AD | 1.0 | 1.0 | NA | 1.0 | 1.0 |
| ALZpath pTau-217 | T^Braak I+^ vs. T- | 1.0 | 1.0 | NA | 1.0 | 1.0 |
| ALZpath pTau-217 | T^Braak V+^ vs. rest | 1.0 | 0.69 | NA | 1.0 | 1.0 |
| ALZpath pTau-217 | A+T^Braak I+^ vs. A+T- | 1.0 | 1.0 | NA | 1.0 | 1.0 |
| ALZpath pTau-217 | A+T^Braak V+^ vs. A+ rest | 1.0 | 1.0 | NA | 1.0 | 1.0 |

The *P*-values of DeLong’s comparison between the indicated ROC-analyses are shown. Abbreviations: rest = T^Braak I-IV^ and T-, pTau = phospho-tau

**Supplementary Table S6. Agreement analysis of PET-based and blood-based classifications.**

|  | Cohort | ALZpath pTau-217 | ALZpath pTau-217 + pTau-181 | ALZpath pTau-217 + pTau-231 | pTau-217+^Janssen^ | pTau-217+^Janssen^ + pTau-181 | pTau-217+^Janssen^ + pTau-231 |
| --- | --- | --- | --- | --- | --- | --- | --- |
| T^Braak I+^ vs. T- | TRIAD | 0.57 (0.46, 0.68) | 0.45 (0.33, 0.56) | 0.4 (0.29, 0.51) | 0.57 (0.46, 0.67) | 0.51 (0.41, 0.61) | 0.47 (0.36, 0.57) |
| T^Braak V+^ vs. rest | TRIAD | 0.63 (0.51, 0.74) | 0.54 (0.43, 0.65) | 0.5 (0.39, 0.6) | 0.72 (0.61, 0.83) | 0.63 (0.52, 0.74) | 0.59 (0.48, 0.69) |
| A+T^Braak I+^ vs. A+T- | TRIAD | 0.44 (0.26, 0.62) | 0.39 (0.19, 0.59) | 0.43 (0.23, 0.62) | 0.50 (0.31, 0.70) | 0.47 (0.27, 0.67) | 0.38 (0.17, 0.59) |
| A+T^Braak V+^ vs. A+ rest | TRIAD | 0.60 (0.43, 0.74) | 0.49 (0.35, 0.63) | 0.44 (0.30, 0.58) | 0.60 (0.44, 0.75) | 0.52 (0.38, 0.66) | 0.50 (0.36, 0.63) |
| T^Braak I+^ vs. T- | WRAP | 0.36 (0.24, 0.48) | 0.25 (0.15, 0.35) | 0.18 (0.091, 0.28) | NA | NA | NA |
| T^Braak V+^ vs. rest | WRAP | 0.60 (0.39, 0.82) | 0.29 (0.13, 0.44) | 0.19 (0.091, 0.3) | NA | NA | NA |
| A+T^Braak I+^ vs. A+T- | WRAP | 0.23 (0.042, 0.42) | 0.14 (-0.034, 0.32) | 0.072 (-0.10, 0.25) | NA | NA | NA |
| A+T^Braak V+^ vs. A+ rest | WRAP | 0.61 (0.39, 0.83) | 0.4 (0.20, 0.59) | 0.19 (0.054, 0.34) | NA | NA | NA |

Cohen’s Kappa coefficient and 95% confidence intervals of indicated agreement analyses for TRIAD and WRAP are shown. Abbreviations: rest = T^Braak I-IV^ and T-, pTau = phospho-tau

**
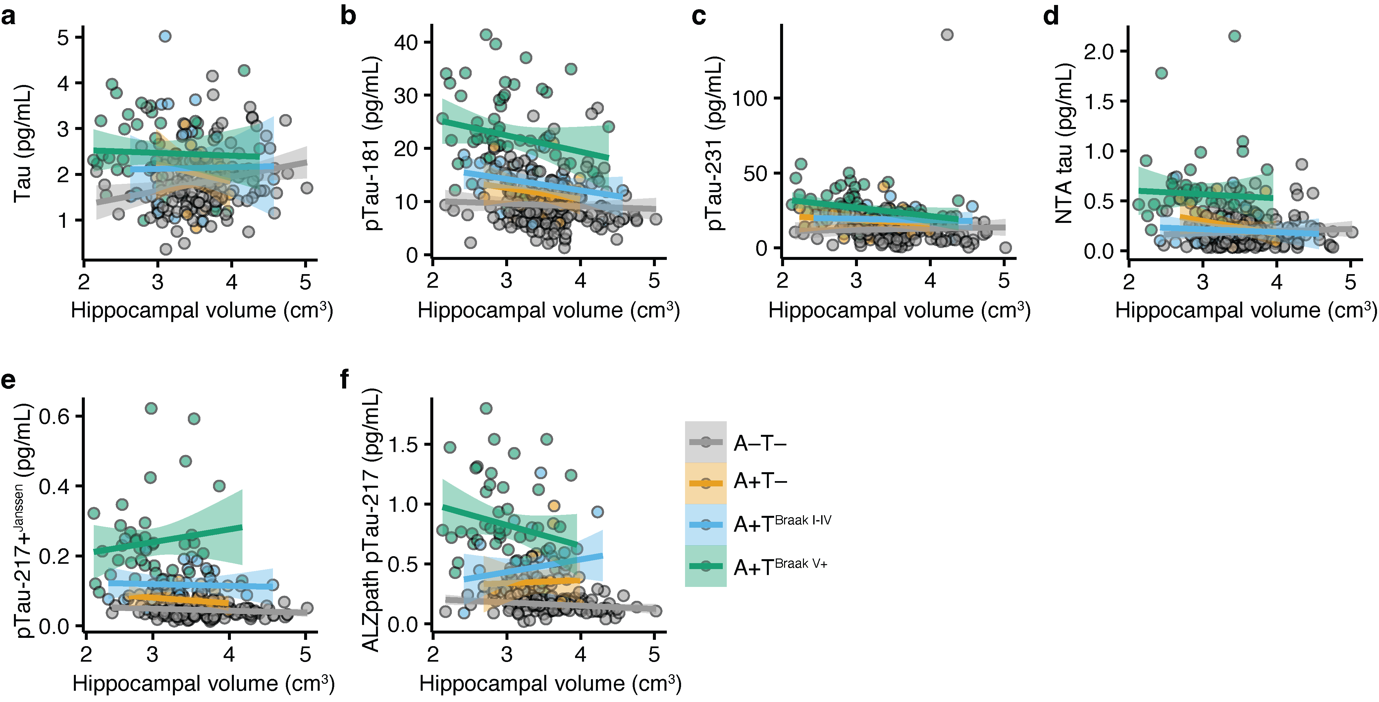
**

**Supplementary Figure S1. Correlation analysis of tau analytes with hippocampal volume.** Pearson correlation analysis of total tau (a), pTau-181 (b), pTau-231 (c), NTA tau (d), pTau-217+^Janssen^ (e), and ALZpath pTau-217 (f) in A-T-, A+T-, A+T^Braak I-IV^, and A+T^Braak V+^ participants of the TRIAD cohort. *P*-values and correlation coefficients are provided in the Supplementary Table S2. The shaded areas correspond to the 95% confidence intervals. pTau = phospho-tau, NTA tau = N-terminal tau.


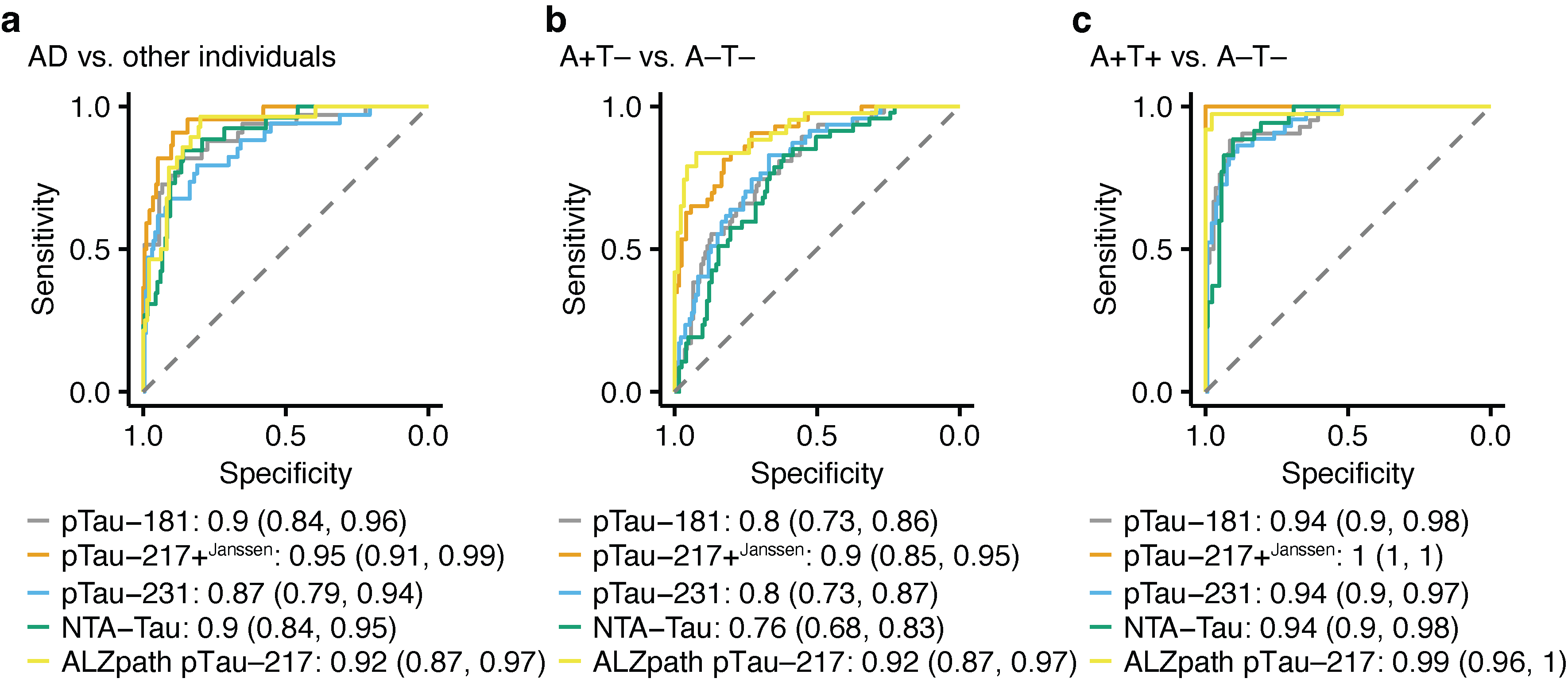


**Supplementary Figure S2. Discrimination of A/T status by blood biomarkers in TRIAD.** Receiver operator characteristic (ROC) analyses to identify individuals with AD vs. non-AD (a), A+T- vs. A-T- (b), and A+T+ vs. A-T- (c). The ROC models include biological sex, age, and APOE ε4 status as covariates. Area under the curves (AUC) and 95% confidence intervals are shown. Dashed line represents AUC of 0.5. pTau = phospho-tau, NTA tau = N-terminal tau.

**
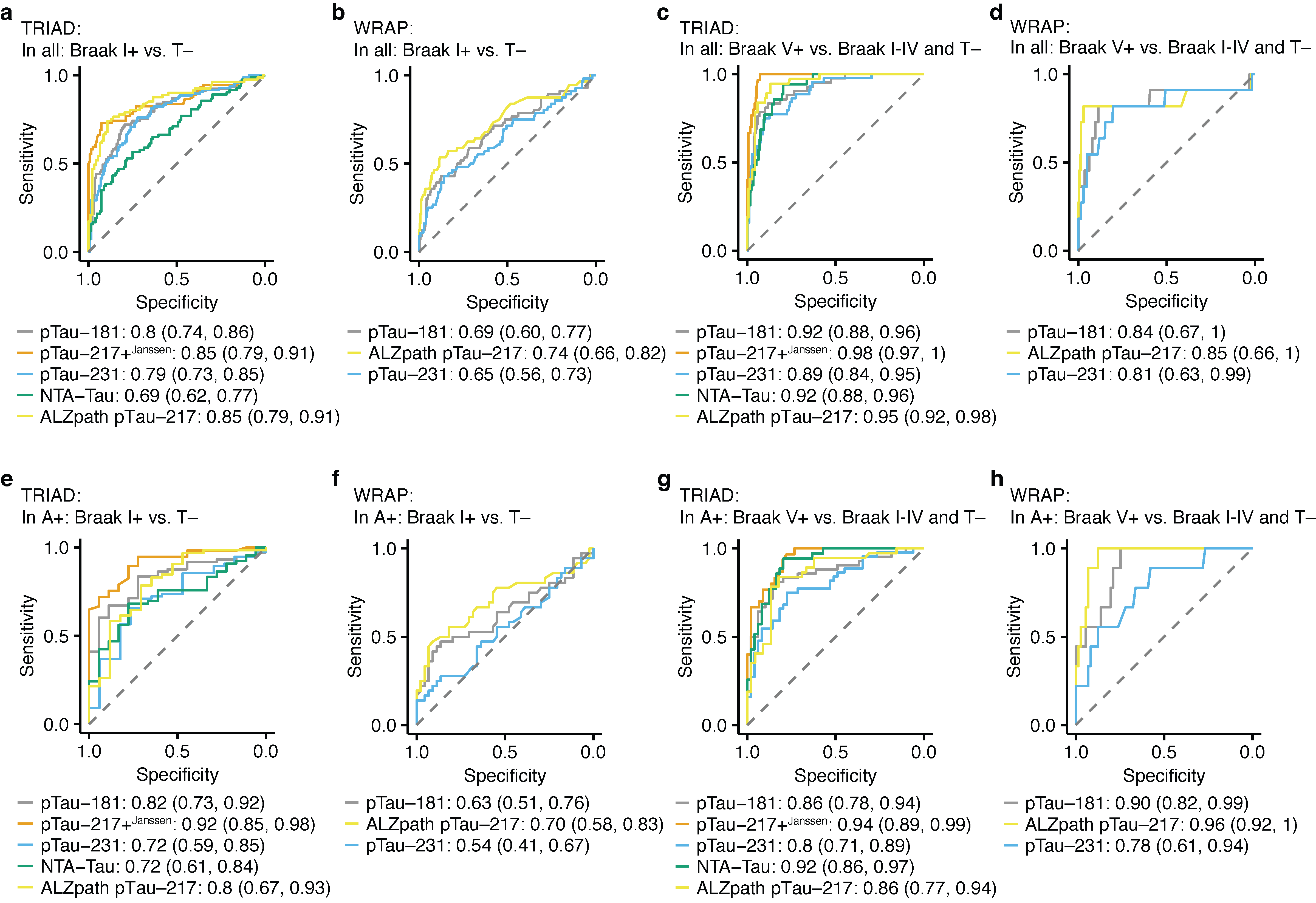
**

**Supplementary Figure S3. Discrimination of tau accumulation in different Braak stages by blood tau biomarkers in TRIAD and WRAP without covariates.** Receiver operator characteristic (ROC) analyses to identify T^Braak I+^ individuals without preselection in TRIAD (a) and WRAP (b), T^Braak V+^ individuals without preselection in TRIAD (c) and WRAP (d), T^Braak I+^ individuals in Aβ+ participants in TRIAD (e) and WRAP (f), T^Braak V+^ individuals in Aβ+ participants in TRIAD (g) and WRAP (h). pTau-181 (pg/mL), pTau-217+^Janssen^ (pg/mL), ALZpath pTau-217 (pg/mL), pTau-231 (pg/mL), and NTA tau (pg/mL) were tested in TRIAD. pTau-181 (pg/mL), ALZpath pTau-217 (pg/mL), pTau-231 (pg/mL) were tested in WRAP. Area under the curve (AUC) and confidence intervals are shown in the figure. Area under the curves (AUC) and 95% confidence intervals are shown. Dashed line represents AUC of 0.5. pTau = phospho-tau, NTA tau = N-terminal tau.


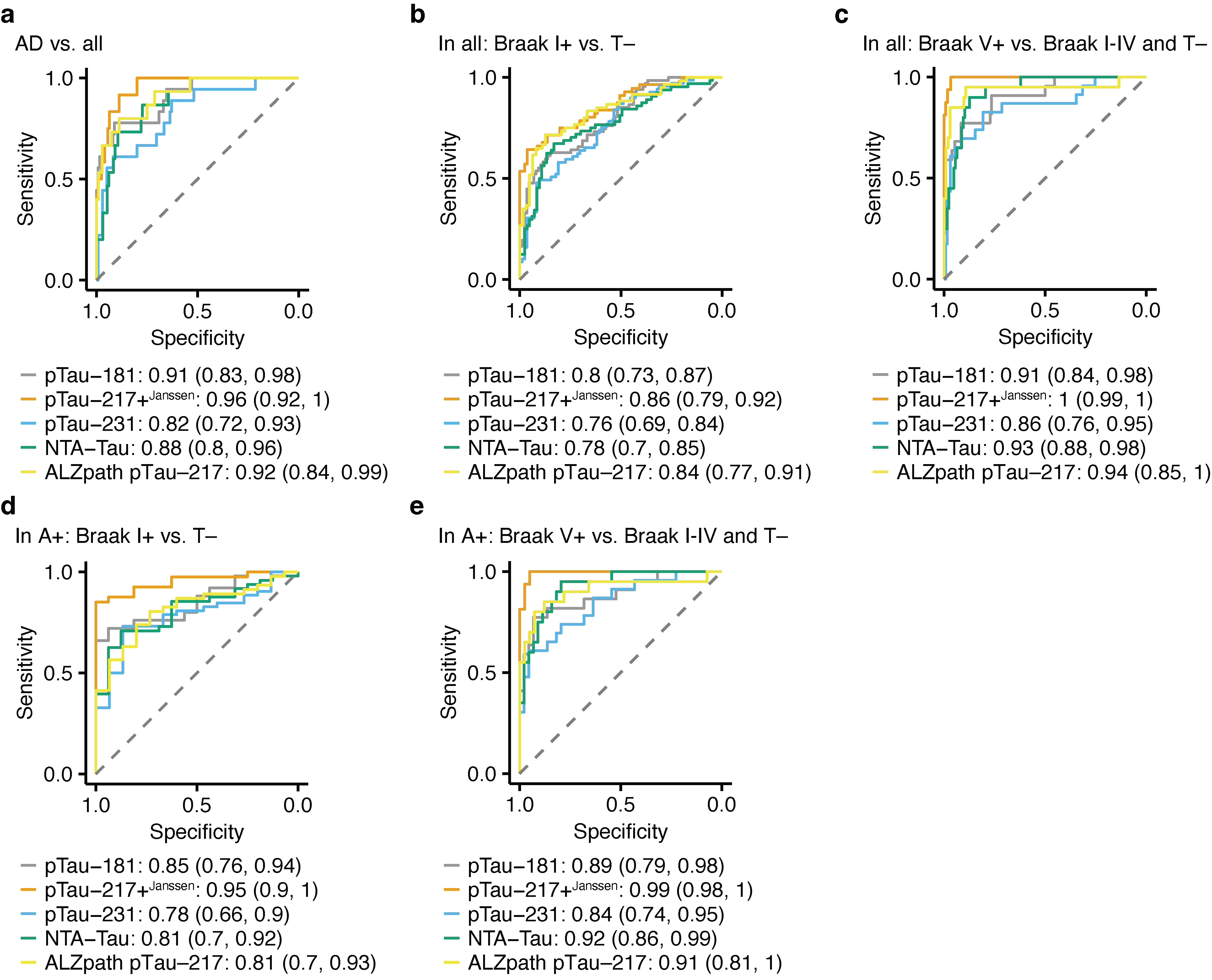


**Supplementary Figure S4. Discrimination of tau accumulation in different Braak stages by blood tau biomarkers in TRIAD participants older than 65 years.** TRIAD participants older than 65 years were analysed (A-T-: n = 110, mean age = 72, standard deviation (SD) age = 4.8; A+T-: n = 22, mean age = 73, SD age = 5.1; A+T^Braak I–IV^: n = 35, mean age = 74, SD age = 5.0; A+T^Braak V+^: n = 35, mean age = 72, SD age = 4.0). Receiver operator characteristic (ROC) analyses to identify AD vs. non-AD (a), T^Braak I+^ individuals without preselection in TRIAD (b), T^Braak V+^ individuals without preselection in TRIAD (c), T^Braak I+^ individuals in Aβ+ participants in TRIAD (d), T^Braak V+^ individuals in Aβ+ participants in TRIAD (e). pTau-181 (pg/mL), pTau-217+^Janssen^ (pg/mL), ALZpath pTau-217 (pg/mL), pTau-231 (pg/mL), and NTA tau (pg/mL) were tested in TRIAD. Area under the curve (AUC) and confidence intervals are shown in the figure. The ROC models include biological sex, age, and APOE ε4 status as covariates. Area under the curves (AUC) and 95% confidence intervals are shown. Dashed line represents AUC of 0.5. pTau = phospho-tau, NTA tau = N-terminal tau.


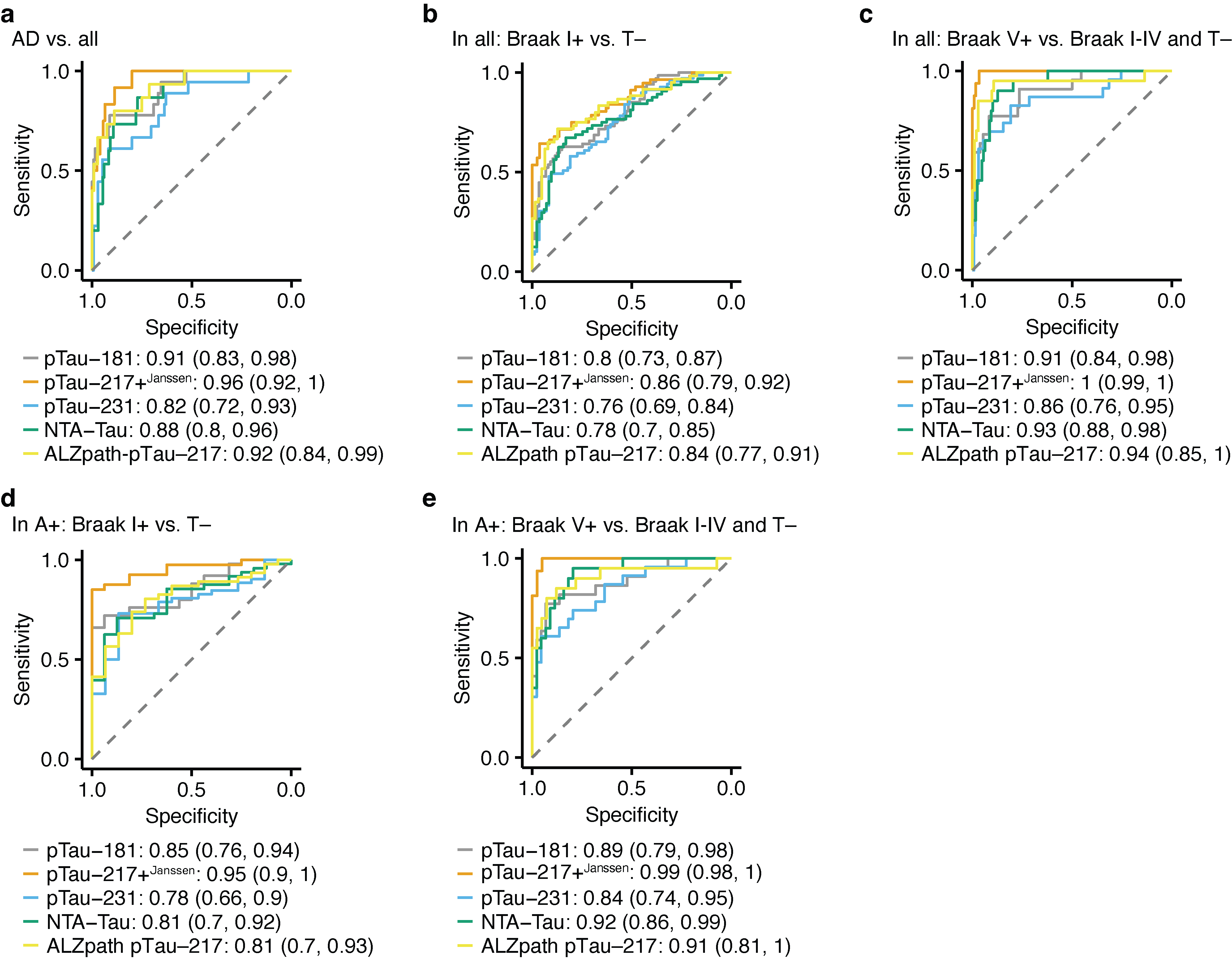


**Supplementary Figure S5. Discrimination of tau accumulation in different Braak stages by blood tau biomarkers in TRIAD participants with MCI and mild AD.** TRIAD participants with MCI and mild AD were analysed (A-T-: n = 18, mean age = 70, SD age = 10.6; A+T-: n = 9, mean age = 72, SD age = 6.6; A+T^Braak I–IV^: n = 18, mean age = 71, SD age = 9.5; A+T^Braak V+^: n = 34, mean age = 71, SD age = 6.1). Receiver operator characteristic (ROC) analyses to identify AD vs. non-AD (a), T^Braak I+^ individuals without preselection in TRIAD (b), T^Braak V+^ individuals without preselection in TRIAD (c), T^Braak I+^ individuals in Aβ+ participants in TRIAD (d), T^Braak V+^ individuals in Aβ+ participants in TRIAD (e). pTau-181 (pg/mL), pTau-217+^Janssen^ (pg/mL), ALZpath pTau-217 (pg/mL), pTau-231 (pg/mL), and NTA tau (pg/mL) were tested in TRIAD. Area under the curve (AUC) and confidence intervals are shown in the figure. The ROC models include biological sex, age, and APOE ε4 status as covariates. Area under the curves (AUC) and 95% confidence intervals are shown. Dashed line represents AUC of 0.5. pTau = phospho-tau, NTA tau = N-terminal tau.


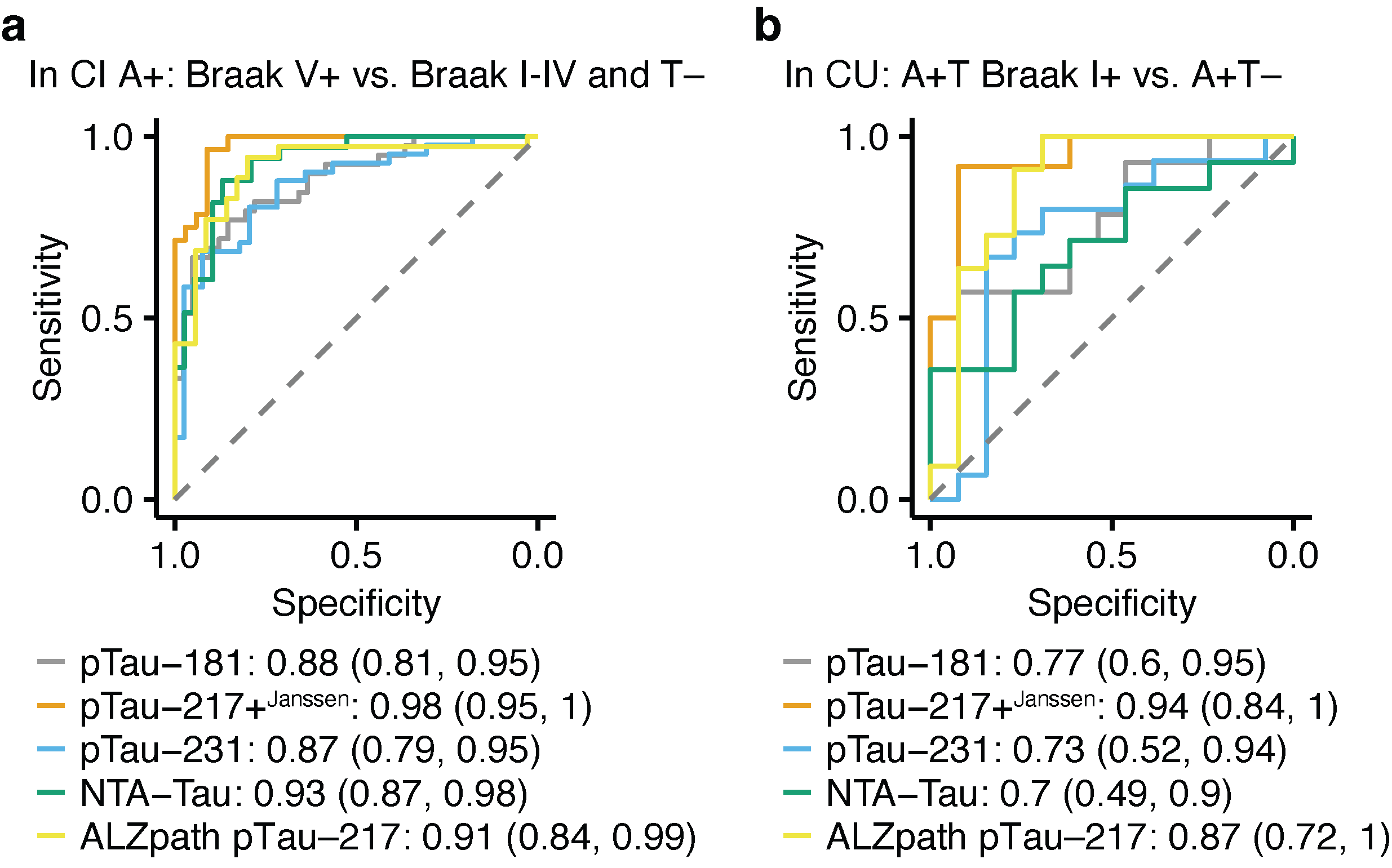


**Supplementary Figure S6. Discrimination of tau accumulation by blood tau biomarkers in TRIAD CI and CU participants.** Receiver operator characteristic (ROC) analyses to (a) identify CI A+T^Braak V+^ (n = 41) vs. CI A+T^Braak I-IV^ and CI A+T- individuals (n = 41; A), and (b) CU A+T^Braak I+^ (n = 16) vs. CU A+T- (n = 14) individuals. pTau-181 (pg/mL), pTau-217+^Janssen^ (pg/mL), ALZpath pTau-217 (pg/mL), pTau-231 (pg/mL), and NTA tau (pg/mL) were tested in TRIAD. Area under the curve (AUC) and confidence intervals are shown in the figure. The ROC models include biological sex, age, and APOE ε4 status as covariates. Area under the curves (AUC) and 95% confidence intervals are shown. Dashed line represents AUC of 0.5. pTau = phospho-tau, NTA tau = N-terminal tau.
